# A novel technique using integral transforms and residual functions for nonlinear partial fractional differential equations involving Caputo derivatives

**DOI:** 10.1371/journal.pone.0313860

**Published:** 2024-12-19

**Authors:** Zareen A. Khan, Muhammad Bilal Riaz, Muhammad Imran Liaqat, Ali Akgül

**Affiliations:** 1 Department of Mathematical Sciences, College of Science, Princess Nourah bint Abdulrahman University, Riyadh, Saudi Arabia; 2 IT4Innovations, VSB-Technical University of Ostrava, Ostrava, Czech Republic; 3 Department of Computer Science and Mathematics, Lebanese American University, Byblos, Lebanon; 4 Abdus Salam School of Mathematical Sciences, Government College University, Lahore, Pakistan; 5 Department of Mathematics, Art and Science Faculty, Siirt University, Siirt, Istanbul, Turkey; 6 Department of Computer Engineering, Biruni University, Istanbul, Turkey; 7 Department of Mathematics, Mathematics Research Center, Near East University, Nicosia /Mersin, Turkey; University of Dhaka, BANGLADESH

## Abstract

Fractional nonlinear partial differential equations are used in many scientific fields to model various processes, although most of these equations lack closed-form solutions. For this reason, methods for approximating solutions that occasionally yield closed-form solutions are crucial for solving these equations. This study introduces a novel technique that combines the residual function and a modified fractional power series with the Elzaki transform to solve various nonlinear problems within the Caputo derivative framework. The accuracy and effectiveness of our approach are validated through analyses of absolute, relative, and residual errors. We utilize the limit principle at zero to identify the coefficients of the series solution terms, while other methods, including variational iteration, homotopy perturbation, and Adomian, depend on integration. In contrast, the residual power series method uses differentiation, and both approaches encounter difficulties in fractional contexts. Furthermore, the effectiveness of our approach in addressing nonlinear problems without relying on Adomian and He polynomials enhances its superiority over various approximate series solution techniques.

## 1 Introduction

Fractional calculus (FrC) is a strong tool for modeling and evaluating systems with recollection and hereditary features. It extends classical calculus to non-integer orders. The history of FrC spans over three centuries, evolving from theoretical curiosity to practical applications in various fields. Here is a detailed historical overview [1].

i. 17th Century
  - 1695: FrC has its roots in a letter that Gottfried Wilhelm Leibniz wrote to Guillaume de l’Hôpital. Leibniz, one of the co-founders of calculus, posed the question about the meaning of a derivative of order $\frac{1}{2}$, sparking initial interest in the concept.
ii. 18th and 19th Centuries
  - 1730: Alexis Clairaut made one of the earliest references to fractional differentiation in a paper on vibrating strings.
  - 1832: In his work on heat conduction, Joseph Fourier proposed expanding the notion of differentiation to fractional orders.
  - 1832: Joseph Liouville formalized the idea of fractional integration and differentiation, providing a more rigorous mathematical framework. He introduced FrC in a systematic way, leading to the development of the Liouville fractional integral and derivative.
  - 1867: Bernhard Riemann expanded on Liouville’s work, contributing to what is now known as the Riemann-Liouville FrC.
iii. 20th Century
  - 1930s-1940s: Paul Lévy and other mathematicians explored fractional processes in stochastic systems, laying the groundwork for applications in various fields, including physics and finance.
  - 1960s-1970s: The work of Kenneth S. Miller and Bertram Ross, as well as Arthur Erdélyi, helped to expand the theoretical foundations of FrC and highlight its potential applications in solving physical problems.
iv. Modern Developments
  - 1980s-Present: FrC became well-known because of its uses in physics, engineering, and other fields of study. The development of digital computers and numerical methods enabled the practical application of fractional differential equations (FDEs) to real-world problems. Researchers began applying FrC to model complex phenomena such as viscoelasticity, anomalous diffusion, and turbulence.

FrC is required for several reasons, the most prominent being that it offers a more precise and thorough framework for characterizing and evaluating complex systems and events. Here are some reasons why FrC is necessary [2, 3]:

i. Integer-order calculus alone is insufficient to accurately characterize the behaviors of many real-world systems. FrC offers a more adaptable and multipurpose modeling tool for these intricate systems. Examples include viscoelastic materials, biological systems with memory effects, and anomalous diffusion processes.
ii. FrC makes it possible to simulate memory-effect systems in which there is a non-trivial dependence between the current and previous states. These kinds of systems are widespread in many disciplines, including as engineering, biology, and physics. Fractional derivatives and integrals capture these memory effects more accurately than integer-order derivatives and integrals.
iii. Many diffusion processes observed in nature do not follow classical Brownian motion. Instead, they exhibit anomalous diffusion, where the mean squared displacement does not scale linearly with time. FrC provides a natural framework for describing and analyzing anomalous diffusion processes.
iv. FrC is closely related to fractal geometry, which deals with objects that have complex, self-similar structures. Fractal phenomena appear in natural and artificial systems, such as coastlines, snowflakes, and financial markets. FrC helps in quantifying and understanding the fractal properties of such systems.
v. FrC is used to build filters, controllers, and estimators for systems with non-local and non-linear behaviors in signal processing and control theory. For instance, as compared to conventional integer-order controllers, fractional-order controllers provide advantages in terms of resilience, stability, and performance.
vi. FrC has been applied to optimization problems and machine learning algorithms, where it helps in designing more efficient optimization techniques and models that capture long-range dependencies and non-linear behaviors more accurately.

System memory effects are closely related to fractional-order derivatives. The ability of a system to remember details about its previous states or inputs, which affects its current behavior, is known as the memory effect. Fractional-order derivatives capture this memory effect by incorporating past states or inputs into the differential equation. In traditional integer-order calculus, derivatives describe how a function changes at a single point in time without considering its history. However, in FrC, the derivatives describe how a function changes over a range of past times, reflecting the memory effect of the system. The memory effect arises from the fact that fractional-order derivatives are non-local operators, meaning they depend on the entire history of the function rather than just its value at the current time. This property allows fractional-order derivatives to capture long-range dependencies, non-local interactions, and complex dynamic behaviors in systems.

Unlike integer-order derivatives, fractional-order derivatives have various definitions, such as Atangana-Baleanu [4], Katugampola fractional derivative [5], Caputo [6] generalized derivative [7], and Hadamard fractional derivative [8]. For further information, interested readers may refer to [9–12], where additional fractional derivatives are discussed.

The Grünwald-Letnikov derivative essentially extends the ordinary derivative into a fractional derivative. It was first introduced by Aleksey Vasilievich Letnikov in 1868 and Anton Karl Grünwald in 1867, respectively [13]. It is as follows:

DλςΩ(λ)=limΘ→01Θς∑ξ=0∞(-1)ξ(ςξ)Ω(λ-ξΘ),

where ξ∈N is as

(ςξ)=ς(ς-1)(ς-2)(ς-3)…(ς-ξ+1)ξ!=Γ(ς+1)ξ!Γ(ς-ξ+1).

Riemann-Liouville fractional derivatives (RLFDs) are novel fractional-order derivatives that were defined by Riemann in 1847 [14]. It has the following definition:

$$\begin{array}{c} D_{\lambda }^{\varsigma }\Omega \left(\lambda \right)=\{\begin{array}{cc} \frac{1}{\Gamma (a-\varsigma )}\frac{d^{a}}{d\lambda^{a}}\int_{0}^{\lambda }{\left(\lambda -\zeta \right)}^{a-\varsigma -1}\Omega \left(\zeta \right)d\zeta , & \text{if}\;a-1\leq \varsigma <a,\\ \frac{d^{a}}{d\lambda^{a}}\Omega \left(\lambda \right), & \text{if}\;\varsigma =a. \end{array} \end{array}$$

The establishment of the Caputo fractional derivative (Cap-FrD) dates back to 1967 [15], when RLFD proved to be inefficient for modeling and explaining some complex events.

$$\begin{array}{c} D_{\lambda }^{\varsigma }\Omega \left(\lambda \right)=\{\begin{array}{cc} \frac{1}{\Gamma (a-\varsigma )}\int_{0}^{\lambda }{\left(\lambda -\zeta \right)}^{a-\varsigma -1}\Omega^{a}\left(\zeta \right)d\zeta , & \text{if}\;a-1\leq \varsigma <a,\\ \frac{d^{a}}{d\lambda^{a}}\Omega \left(\lambda \right), & \text{if}\;\varsigma =a, \end{array} \end{array}$$

where $a-1\leq \varsigma <a\in Z^{+}$.

The expression $\frac{W\lambda^{-a}}{\Gamma (1-\varsigma )}$ represents the RLFD of a constant W. Therefore, one of Cap-FrD’s advantages is that the derivative of a constant is zero, just like in an ordinary derivative.

The Caputo fractional operator is often preferred over other fractional operators for several reasons [16]:

i. The Caputo derivative allows for the use of initial conditions in the same form as those used for integer-order differential equations. This makes it more intuitive and easier to apply to physical and engineering problems.
ii. The Caputo derivative is more suitable for modeling real-world phenomena because it provides a more straightforward physical interpretation. For example, it is often used in viscoelasticity and anomalous diffusion.
iii. The Caputo derivative is compatible with classical methods of solving differential equations, such as the Laplace transform. This compatibility simplifies the process of finding analytical solutions.
iv. Unlike some other fractional derivatives, the Caputo derivative has a non-singular kernel, which can be advantageous in certain applications
v. The Caputo derivative of a constant is zero, which aligns with the classical derivative and simplifies the analysis of differential equations.

Complex behaviors found in many natural occurrences are beyond the scope of linear models or traditional integer-order differential equations. Nonlinear partial fractional differential equations (NPFDEs) provide a more realistic framework for modeling these phenomena, accounting for non-local interactions, memory effects, and intricate dynamics. NPFDEs combine the features of partial differential equations (PDEs), which describe systems involving multiple independent variables, and FrC, which involves derivatives of non-integer order. NPFDEs find applications in various fields due to their ability to model complex phenomena exhibiting non-locality, memory effects, and nonlinearity simultaneously. Here are some notable applications [17, 18]:

i. NPFDEs are used to simulate a wide range of physical phenomena in complex media, including heat transfer, fluid flow, and electromagnetic wave propagation. For example, they can describe the behavior of fluids with non-Newtonian properties or materials with fractal geometries.
ii. NPFDEs are applied in biological and medical modeling to understand processes like diffusion, reaction-diffusion systems, and tumor growth. They help in studying the transport of nutrients and drugs in biological tissues and the dynamics of cellular populations.
iii. NPFDEs find applications in engineering disciplines such as mechanical, civil, and chemical engineering. They are used to model and analyze complex systems, including structural mechanics, fluid dynamics, and chemical reactions.
iv. NPFDEs are employed in financial mathematics to model the dynamics of financial markets and assets. They help in understanding price movements, volatility clustering, and risk management strategies in markets with long-range dependencies and memory effects.
v. NPFDEs are used in geophysical modeling to study phenomena such as seismic wave propagation, groundwater flow, and heat transfer in the earth’s crust. They aid in understanding natural hazards like earthquakes and tsunamis.

It is crucial to solve NPFDEs because they offer the mathematical foundation for characterizing and forecasting the behavior of complex systems in a variety of domains. The main kinds of solutions are analytical solutions and approximate solutions. An analytical solution is an exact, closed-form expression that precisely satisfies a given mathematical problem or equation. It typically involves standard mathematical functions (e.g., polynomials, exponentials, trigonometric functions) and can be written explicitly. An approximate solution is an estimated solution to a mathematical problem, obtained using numerical methods, iterative techniques, or approximations. It is not an exact expression but provides a solution that is sufficiently close to the exact solution within a specified level of accuracy. Therefore, comprehending the solutions of NPFDEs holds significant importance in deciphering the underlying essence of physical phenomena. The solutions to NPFDEs are extremely important for several reasons:

i. Fractional derivatives capture anomalous transport phenomena observed in various systems, such as subdiffusion and superdiffusion. Solutions to NPFDEs help understand and predict the behavior of particles or quantities undergoing such anomalous transport, which is essential in fields like physics, biology, and material science.
ii. Fractional derivatives encode memory effects, allowing NPFDEs to capture long-range dependencies in systems. Solutions to NPFDEs provide insights into the long-term behavior and evolution of dynamic systems, enabling predictions beyond the scope of classical differential equations (DEs).
iii. NPFDEs arise in control theory, optimization problems, and decision-making processes. Understanding their solutions helps in designing better control strategies, optimizing processes, and making informed decisions in complex systems.
iv. FrC is increasingly used in signal processing applications due to its ability to capture non-local and nonstationary features in signals. Solutions to NPFDEs contribute to advanced signal processing techniques such as denoising, feature extraction, and time-series analysis.
v. NPFDEs arise in various engineering applications, including fluid dynamics, heat transfer, electromagnetics, and structural mechanics. Solving these equations aids in designing and optimizing engineering systems with improved accuracy and efficiency.
vi. NPFDEs present interesting mathematical challenges due to their non-local, non-convex, and non-smooth nature. Developing numerical methods and analytical techniques for solving these equations contributes to advancements in mathematical analysis and computational science.

NPFDEs include nonlinear terms and fractional derivatives, making them challenging to solve and analyze. The majority of NPFDEs lack exact solutions, so techniques offering approximate solutions have become indispensable for addressing such challenges. Several methods that establish approximate solutions, including the operational matrix [19], the Haar wavelet collocation [20], the differential transforms [21], the reproducing kernel [22], the Adomian decomposition technique (ADT) with Aboodh transform [23], and the homotopy perturbation approach (HPA) [24], Adomian decomposition with general transform [25], have been proposed for NPFDEs in the past few years. For more details about methods, see [26–30].

The Laplace transform (L-T), developed in the 18th century by Pierre-Simon Laplace, has been a cornerstone in solving DEs and analyzing systems in mathematics, physics, and engineering. However, the Elzaki transform (E-T) offers certain advantages that make it more effective in some applications, despite the Laplace transform’s longer history and widespread use.

The E-T offers several advantages over the L-T [31, 32].

i. Unlike the L-T, the E-T preserves the scales and units of the original function. This can be particularly useful in engineering and physical applications where maintaining the original units is important.
ii. The E-T has a duality relationship with the L-T, which means it can be used as an auxiliary method to the L-T. This duality can simplify the process of solving complex DEs by providing an alternative approach.
iii. The E-T offers a more efficient, straightforward, and flexible approach to solving FDEs than the Laplace transform. Its ability to handle fractional derivatives naturally, incorporate initial conditions easily, and avoid complex inversions makes it a superior tool in the realm of FC.
iv. The E-T is more effective in solving ordinary DEs with variable coefficients.
v. It provides a straightforward method for incorporating initial conditions, which can sometimes be more cumbersome with the L-T.

The E-T is superior to the Sumudu transform (S-T) for solving DEs, as we verify from the following two examples. The S-T cannot solve these problems, but the E-T solves them in a straightforward way.

$$\begin{array}{c} \tau^{2}f^{\prime \prime }\left(\tau \right)+4\tau f^{\prime }+2f=12\tau^{2},\;f\left(0\right)=f^{\prime }\left(0\right)=0. \end{array}$$ (1)

By applying S-T to the above system, get as

$$\begin{array}{c} v^{2}G^{\prime \prime }\left(v\right)+4vG^{\prime }\left(v\right)+2G\left(v\right)=24v^{2}. \end{array}$$ (2)

Since Eqs (1) and (2) are identical, the S-T cannot solve the given system.

Now if we apply the E-T to Eq (1), we have the following:

$$\begin{array}{c} v^{4}\frac{d^{2}}{dv}(\frac{G(v)}{v^{2}})+4v^{2}(\frac{G(v)}{v})-4v(\frac{G(v)}{v})+2G\left(v\right)=24v^{4}\;or\;G^{\prime \prime }=24v^{2}. \end{array}$$

So,

$$\begin{array}{c} G\left(v\right)=2v^{4}+r_{1}v+r_{2}. \end{array}$$

Using the conditions to find $r_{1}=r_{2}=0$, then G(v) = 2v^4^.

We obtain the solution in the following form by applying the inverse E-T: f(*τ*) = *τ*^2^.

Consider the third-order non-constant coefficient DE:

$$\begin{array}{c} \tau^{2}f^{\prime \prime \prime }+6\tau f^{\prime \prime }+6f^{\prime }=60\tau^{2},\;f\left(0\right)=f^{\prime }\left(0\right)=f^{\prime \prime }\left(0\right)=0. \end{array}$$ (3)

If we use the S-T and the initial conditions (I.Cs), we find that:

$$\begin{array}{c} v^{2}G^{\prime \prime }\left(v\right)+4vG^{\prime }+2G\left(v\right)=120v^{2}, \end{array}$$

which is the same Eq (3), and again, the S-T fails to solve this equation. Now, by applying the E-T to Eq (3) and making use of the I.Cs, we get:

$$\begin{array}{c} G^{\prime \prime }=120v^{3}. \end{array}$$

So,

$$\begin{array}{c} G\left(v\right)=6v^{5}+r_{1}v+r_{2}. \end{array}$$

By using the I.Cs, we find: $r_{1}=r_{2}=0$. Therefore, G(v) = 6v^5^.

Through the use of the inverse E-T, we arrive to the following solution form: f(*τ*) = *τ*^3^.

Fractional gas dynamics models offer a thorough and precise framework for simulating various intricate physical phenomena, making them an important field of research. Traditional gas dynamics often rely on integer-order DEs, which may not capture the full complexity of certain systems, especially those with memory effects, non-local interactions, or anomalous diffusion.

This study uses the following type of fractional gas dynamics equation (FrGDE) [33]:

$$\begin{array}{c} D_{\lambda }^{\varsigma }\Omega \left(\chi ,\lambda \right)=-\Omega \left(\chi ,\lambda \right)D_{\chi }\Omega \left(\chi ,\lambda \right)+G\Omega \left(\chi ,\lambda \right)\left(1-\Omega \left(\chi ,\Omega \right)\right)+B\left(\chi ,\lambda \right), \end{array}$$

under the I.C listed below:

$$\begin{array}{c} \Omega (\chi ,0)=A(\chi ), \end{array}$$

where *χ* ≥ 0, *ς* is fractional derivative with 0<ς≤1,G is an appropriate constant and *B*(*χ*, λ) represent the basis term.

Researchers have shown keen interest in finding solutions for FrGDEs. For example, Kumar et al. [34] established approximate solutions to time FrGDEs utilizing the perturbation transform approach. In [35], the authors utilized the homotopy analysis approach (HAA) to establish solutions for FrGDEs. Das and Kumar [36] employed the differential transform method to derive approximate solutions for FrGDEs. The authors [37] utilized shifted Legendre polynomials to find solutions for the FrGDEs. In [38], the authors found approximate solutions for the time FrGDEs. Jebreen and Cattani [39] used the collocation method to extract solutions for the FrGDEs.

The fractional Swift-Hohenberg equation (FrSHE) is utilized across a spectrum of engineering and scientific fields, including laser studies, biology, physics, hydrodynamics, and fluid dynamics. It is essential for comprehending the generation of patterns in fluid layers constrained by horizontal well-conducting barriers. The modeling of pattern formation involves tackling diverse challenges like pattern selection, the impact of noise on bifurcations, defect dynamics, and the occurrence of spatiotemporal chaos, all of which heavily rely on this equation [40]. The standard expression of the FrSHE is given by:

$$\begin{array}{c} D_{\lambda }^{\varsigma }\Omega \left(\chi ,\lambda \right)=Q\Omega \left(\chi ,\lambda \right)-{\left(1+\nabla^{2}\right)}^{2}\Omega \left(\chi ,\lambda \right)-\Omega^{3}\left(\chi ,\lambda \right), \end{array}$$

where *χ* ∈ *R*, λ > 0, *ς* is fractional derivative with 0 < *ς* ≤ 1, Q is bifurcation parameter, and Ω(*χ*, λ) is a scaler function of *χ* and λ defined on the line or the plane.

Researchers have also shown keen interest in finding solutions for FrSHE. For example, Nonlaopon et al. [41] used the Adomian method to solve the time-fractional FrSHEs. Li and Pang [42] used the iterative method to find solutions to time-fractional FrSHEs. Veeresha et al. [43] applied the q-homotopy technique to find solutions to time-fractional FrSHEs. Jani and Singh [44] utilized the HPA to solve fractional FrSHEs. Pavani and Raghavendra [45] used natural transform with ADT to solve these fractional equations.

Various fields within natural science, such as quantum optics, theoretical biology, circuit theory, chemical physics, solid-state physics, and more, utilize the fractional Fokker-Planck equation (FrFPE). Originally introduced by Fokker and Planck to illustrate Brownian motion [46], the FrFPE applies the general framework outlined below [47]:

$$\begin{array}{c} D_{\lambda }^{\varsigma }\Omega \left(\chi ,\lambda \right)=\left(-D_{\chi }W\left(\chi \right)+D_{\chi \chi }Z\left(\chi \right)\right)\Omega \left(\chi ,\lambda \right), \end{array}$$

with I.C:

$$\begin{array}{c} \Omega (\chi ,0)=H(\chi ),\;\chi \in R, \end{array}$$

where *Z*(*χ*) > 0 is the diffusion factor and *W*(*χ*)>0 is the drift factor.

Researchers have also demonstrated a strong need to find solutions for FrFPEs. For instance, Yang et al. [48] employed spectral collocation methods to solve the FrFPEs. He et al. [49] utilized the Fourier transform to find solutions to these equations. The author [50] applied the Laplace iterative method for solving the FrFPEs. Baumann and Stenger [51] established solutions to these equations using the Sinc method. Khan et al. [52] employed a novel series approach to solve the FrFPEs.

All the above approaches used to solve models of FrGDEs, FrFPEs, and FrSHEs entail lengthy computation periods and significant computational requirements. To solve several nonlinear problems within the context of the Cap-FrD, this study employs a novel technique that combines the E-T with modified fractional power series (FPS) and the residual function. Our novel technique, called the Elzaki residual approach (ERA), is used to provide both approximate solutions (App-Ss) and exact solutions (Ex-Ss) for FrGDEs, FrSHEs, and FrFPEs in terms of Cap-FrD. The results obtained from our technique for FrGDEs, FrSHEs, and FrFPE exhibit high agreement with the differential transform method [36], HPA with Aboodh transform [44], and analytical computational scheme [52], respectively. This demonstrates that our technique is a suitable alternative tool for solving nonlinear models. The algorithm of our technique consists of the following steps: In the first step, the given equation is transformed into the E-T space by applying the E-T. As a result, we obtain an algebraic form in the E-T space. In the second phase, the novel FPS in E-T is employed to express the solution of the algebraic equation obtained in the first step in the E-T space. The coefficients of this expansion are determined using residual functions and the limit notion. Subsequently, we solve the problem in the original space by finding the inverse E-T.

We utilized residual error (Res-E), relative error (Rel-E), and absolute error (Abs-E) measurements to validate the accuracy of the App-Ss obtained through our technique for nonlinear problems. ERA offers the advantage of not needing any parameters in the equation, allowing it to solve both weak and highly NPFDEs and avoiding some of the limitations of conventional perturbation approaches. Our technique has proven to be more effective than other series solution methods because it can handle nonlinear problems without relying on any polynomials. Its capability to address nonlinear problems without using polynomials such as Adomian and He makes ERA more efficient than alternative series solution techniques. To find the coefficients of the FPS, we use the limit principle at zero. In contrast, the residual power series approach employs differentiation, while other techniques such as ADT, HPA, and variational approaches rely on integration. Both approaches face challenges in the fractional case. Based on these results, we conclude that our approach is accurate and easy to apply. In the future, we plan to utilize ERA to solve various types of nonlinear fractional models encountered in biological systems and engineering domains.

Our method has certain limitations as well. To obtain the solution in the original space, the ERA requires first finding the E-T of the target equations, followed by performing the inverse E-T. Consequently, for nonhomogeneous equations, the source functions need to be piecewise continuous and of exponential order, with the inverse E-T being required to exist after computations. Additionally, this method assumes that the Caputo derivative adheres to the semigroup property.

The subsequent sections are organized as follows: Section 2: Preliminary presents the main definitions and results that form the foundation of our study, as established in this manuscript. Section 3: The Algorithm of the ERA for Solving NFPDEs introduces the ERA algorithm and its convergence criteria. In Section 4: Applications of the ERA, we solve three types of NFPDEs using the ERA. Section 5: Graphical and Numerical Results discusses the outcomes obtained in Section 4: Applications of the ERA, analyzing them both graphically and numerically to validate our approach. Section 6: Conclusions summarizes our findings, and finally, Section 7: Future Directions outlines potential avenues for further research.

## 2 Preliminaries

The common definitions and characteristics that we employed throughout this study are all presented in this section.

**Definition 2.1** [53] *The E-T is based on the traditional Fourier integral. The E-T was established by Tarig. M. Elzaki to facilitate the time-domain solution of ordinary and PDEs. It is a very successful approach to solving fractional problems. We examine the functions in a set* Q, *which is described as*:

$$\begin{array}{c} Q=\{\Omega \left(\lambda \right)|\exists P,g_{1},g_{2}>0,|\Omega \left(\lambda \right)<Pe^{\left|\lambda \right|g_{i}}\;if\;\lambda \in {\left(-1\right)}^{\iota }\times \left[0,\infty \right)\}. \end{array}$$

*E-T is constructed with the formulation of*:

$$\begin{array}{c} E_{\varsigma }\left[\Omega \left(\lambda \right)\right]=B\left(q\right)=q\int_{0}^{\infty }\Omega \left(\lambda \right)e^{-\frac{\lambda }{q}}d\lambda ,\;g_{1}\leq q\leq g_{2}. \end{array}$$

**Definition 2.2** [54] *Assume that* Ω(λ) *satisfies the axioms of existence of E-T, then we have*

$$\begin{array}{c} B\left(\chi ,q\right)=\sum_{\iota =0}^{\infty }q^{\iota \varsigma +2}{ℵ}_{\iota }\left(\chi \right). \end{array}$$

**Lemma 1** [55] *Assume that* Ω_1_(*χ*, λ) *and* Ω_2_(*χ*, λ) *satisfies the axioms of existence of E-T*, $E_{\varsigma }\left[\Omega_{1}\left(\chi ,\lambda \right)\right]=B_{1}\left(\chi ,q\right),E_{\varsigma }\left[\Omega_{2}\left(\chi ,\lambda \right)\right]=B_{2}\left(\chi ,q\right)$ *and C*_1_, *C*_2_ *are constants. Then, the following axioms hold*:

*i*. $E_{\varsigma }\left[C_{1}\Omega_{1}\left(\chi ,\lambda \right)+C_{2}\Omega_{2}\left(\chi ,\lambda \right)\right]=C_{1}B_{1}\left(\chi ,q\right)+C_{2}B_{2}\left(\chi ,q\right)$.
*ii*. $E_{\varsigma }^{-1}\left[C_{1}B_{1}\left(\chi ,q\right)+C_{2}B_{2}\left(\chi ,q\right)\right]=C_{1}\Omega_{1}\left(\chi ,\lambda \right)+C_{2}\Omega_{2}\left(\chi ,\lambda \right)$.
*iii*. ${ℵ}_{0}\left(\chi \right)={\text{lim}}_{q\to 0}(\frac{1}{q^{2}}B\left(\chi ,q\right))$.
*iv*. $E_{\varsigma }\left[D_{\lambda }^{\varsigma }\Omega \left(\chi ,\lambda \right)\right]=q^{-\varsigma }B\left(\chi ,q\right)-\sum_{\kappa =0}^{s-1}q^{\left(2+\kappa -\varsigma \right)}D_{\lambda }^{\left(\kappa \right)}\left(\chi ,0\right),\;s-1<\varsigma \leq s$.
*v*. $E_{\varsigma }\left[D_{\lambda }^{\iota \varsigma }\Omega \left(\chi ,\lambda \right)\right]=q^{-\iota \varsigma }B\left(\chi ,q\right)-\sum_{\kappa =0}^{\iota -1}q^{\left(2+(\kappa -\iota )\varsigma \right)}D_{\lambda }^{\left(\kappa \right)}\left(\chi ,0\right),\;0<\varsigma \leq 1$.

**Theorem 1** [55] *The E-T of* Ω(*χ*, λ) *is* $E_{\varsigma }\left[\Omega \left(\chi ,\lambda \right)\right]=B\left(\chi ,q\right)$ *and FPS is given by* $B\left(\chi ,q\right)=\sum_{\iota =0}^{\infty }q^{\iota \varsigma +2}{ℵ}_{\iota }\left(\chi \right)$, *then we have*

$$\begin{array}{c} {ℵ}_{\iota }\left(\chi \right)=D_{\lambda }^{\iota \varsigma }\Omega \left(\chi ,0\right), \end{array}$$

*where* *ι* = 0, 1, 2, …, *and* $D_{\lambda }^{\iota \varsigma }=D_{\lambda }^{\varsigma }.\;D_{\lambda }^{\varsigma }.\;D_{\lambda }^{\varsigma }\;.\;.\;.\;D_{\lambda }^{\varsigma }\left(\iota -times\right)$.

## 3 The algorithm of the ERA for solving NFPDEs

In this section, we present the main steps for solving NFPDEs using E-T, the residual function, and FPS. We apply these steps to solve NFPDEs in the standard operator structure.

The following steps explain the primary algorithm of our approach for solving Eq (4). By utilizing E-T and FPS with the residual function, the general analytic solution of the nonlinear PDEs in the standard operator structure is provided below:

$$\begin{array}{c} D_{\lambda }^{\varsigma }\Omega \left(\chi ,\lambda \right)+L\left(\Omega \left(\chi ,\lambda \right)\right)+N\left(\Omega \left(\chi ,\lambda \right)\right)=R\left(\chi ,\lambda \right), \end{array}$$ (4)

with the initial condition:

$$\begin{array}{c} \Omega (\chi ,0)=G(\chi ), \end{array}$$ (5)

where $\chi >0,\lambda >0,0<\varsigma \leq 1,D_{\lambda }^{\varsigma }$ is the Cap-FrD of λ(χ,λ),L(λ(χ,λ)) stands for a differential operator that is linear, N(Ω(χ,λ)) for one that is nonlinear, R(χ,λ) for a specified term and G(χ) for the function of *χ*.

Step 1: Applying E-T to Eq (4), using the linear property of E-T and Lemma 1(iv) and making some calculations, we get
  $$\begin{array}{ccc} B(\chi ,q) & = & -q^{\varsigma }(L(B(\chi ,q)))-q^{\varsigma }(N(B(\chi ,q)))+q^{2}G\left(\chi \right)+q^{\varsigma }H\left(\chi ,q\right), \end{array}$$ (6)
  where $E_{\varsigma }\left[\Omega \left(\chi ,\lambda \right)\right]=B\left(\chi ,q\right)$ and $E_{\varsigma }\left[R\left(\chi ,\lambda \right)\right]=H\left(\chi ,q\right)$.
Step 2: Consider the solution of Eq (6) in the following FPS:
  $$\begin{array}{c} B\left(\chi ,q\right)=\sum_{\iota =0}^{\infty }q^{\iota \varsigma +2}{ℵ}_{\iota }\left(\chi \right). \end{array}$$ (7)
Step 3: Obtain the *κ*th-truncated FPS as
  $$\begin{array}{c} B_{\kappa }\left(\chi ,q\right)=\sum_{\iota =0}^{\kappa }q^{\iota \varsigma +2}{ℵ}_{\iota }\left(\chi \right). \end{array}$$ (8)
Step 4: By using the following Lemma 1(iii), we have the following
  $$\begin{array}{c} {ℵ}_{0}\left(\chi \right)=\underset{q\to 0}{\text{lim}}(\frac{1}{q^{2}}B\left(\chi ,q\right))=G\left(\chi \right). \end{array}$$ (9)
  The *κ*th-truncated series becomes as
  $$\begin{array}{c} B_{\kappa }\left(\chi ,q\right)=q^{2}G\left(\chi \right)+\sum_{\iota =1}^{\kappa }q^{\iota \varsigma +2}{ℵ}_{\iota }\left(\chi \right). \end{array}$$ (10)
Step 5: The Elzaki residual function (ERESF) and *κ*th-truncated ERESF are as
  $$\begin{array}{c} ERES\left(\chi ,q\right)=B\left(\chi ,q\right)+q^{\varsigma }(L(B(\chi ,q)))+q^{\varsigma }(N(B(\chi ,q)))-q^{2}G\left(\chi \right)-q^{\varsigma }H\left(\chi ,q\right). \end{array}$$
  $$\begin{array}{c} E{RES}_{\kappa }\left(\chi ,q\right)=B_{\kappa }\left(\chi ,q\right)+q^{\varsigma }(L(B_{\kappa }\left(\chi ,q\right)))+q^{\varsigma }(N(B_{\kappa }\left(\chi ,q\right)))-q^{2}G\left(\chi \right)-q^{\varsigma }H\left(\chi ,q\right). \end{array}$$
Step 6: Determine the solution for ℵ_*ι*_(*χ*) in the following:
  $$\begin{array}{c} \underset{q\to 0}{\text{lim}}(\frac{1}{q^{\kappa \varsigma +2}}E{RES}_{\kappa }\left(\chi ,q\right))=0. \end{array}$$ (11)
Step 7: Substitute the calculated values of ℵ_*κ*_(*χ*) into the truncated series up to the *κ*th term of $B_{\kappa }\left(\chi ,q\right)$ to obtain the *κ*th App-S of Eq (6).
Step 8: Utilize the inverse E-T on $B_{\kappa }\left(\chi ,q\right)$ to derive the *κ*th App-S Ω_*κ*_(*χ*, λ).

The subsequent theorem provides a detailed explanation and establishes the convergence criteria for the revised version of FPS.

**Theorem 2** *Let* $E_{\varsigma }\left[\Omega \left(\chi ,\lambda \right)\right]=B\left(\chi ,q\right)$ *be represented as the novel form of FPS* $B\left(\chi ,q\right)=\sum_{\iota =0}^{\infty }q^{\iota \varsigma +2}{ℵ}_{\iota }\left(\chi \right)$ . *If the following condition is fulfilled*

$$\begin{array}{c} |\frac{1}{q^{2}}E_{\varsigma }[D_{\lambda }^{\left(\iota +1\right)}\Omega \left(\chi ,\lambda \right)|\leq M, \end{array}$$

*then* $R_{\iota }\left(\chi ,q\right)$ *of the novel form of FPS fulfills the following*:

$$\begin{array}{c} |R_{\iota }\left(\chi ,q\right)|\leq q^{(\iota +1)q+2}M. \end{array}$$

*Proof: Take into consideration the following from the FPS*:

$$\begin{array}{c} R_{\iota }\left(\chi ,q\right)=\Omega \left(\chi ,q\right)-\sum_{\kappa =0}^{\iota }q^{\kappa \varsigma +2}{ℵ}_{\kappa }\left(\chi \right). \end{array}$$ (12)

Eq (12) *converts through the application of Theorem 1 as follows*:

$$\begin{array}{c} R_{\iota }\left(\chi ,q\right)=\Omega \left(\chi ,q\right)-\sum_{\kappa =0}^{\iota }q^{\kappa \varsigma +2}D_{\lambda }^{\kappa \varsigma }\Omega \left(\chi ,0\right). \end{array}$$ (13)

*Divide both sides of* Eq (13) *by* $q^{(\iota +1)\varsigma +2}$. *As a result, we have the following*:

$$\begin{array}{c} \frac{1}{q^{(\iota +1)\varsigma +2}}R_{\iota }\left(\chi ,q\right)=\frac{1}{q^{2}}(\frac{1}{q^{(\iota +1)\varsigma }}\Omega \left(\chi ,q\right)-\sum_{\kappa =0}^{\iota }\frac{1}{q^{(\iota +1-\kappa )\varsigma -2}}D_{\lambda }^{\kappa \varsigma }\Omega \left(\chi ,0\right)). \end{array}$$ (14)

*When Lemma 1(v) is applied to* Eq (14), *we obtain*:

$$\begin{array}{c} \frac{1}{q^{(\iota +1)\varsigma +2}}R_{\iota }\left(\chi ,q\right)=\frac{1}{q^{2}}E\left[D_{\lambda }^{(\iota +1)\varsigma }\Omega \left(\chi ,\lambda \right)\right]. \end{array}$$ (15)

*By utilizing the absolute value notation in* Eq (15), *we obtain*:

$$\begin{array}{c} |\frac{1}{q^{(\iota +1)\varsigma +2}}R_{\iota }\left(\chi ,q\right)|=|\frac{1}{q^{2}}E\left[D_{\lambda }^{(\iota +1)\varsigma }\Omega \left(\chi ,\lambda \right)\right]|. \end{array}$$ (16)

*A specific result is obtained by applying the provided condition to* Eq (16).

$$\begin{array}{c} |\frac{1}{q^{(\iota +1)\varsigma +2}}R_{\iota }\left(\chi ,q\right)|\leq M. \end{array}$$

$$\begin{array}{c} -Mq^{(\iota +1)\varsigma +2}\leq R_{\iota }\left(\chi ,q\right)\leq Mq^{(\iota +1)\varsigma +2}. \end{array}$$ (17)

*The required result is obtained from* Eq (17).

$$\begin{array}{c} |R_{\iota }\left(\chi ,q\right)|\leq Mq^{(\iota +1)\varsigma +2}. \end{array}$$

A new series convergence condition has been established as a result of preceding statements or calculations.

## 4 Applications of the ERA

In this section, the effectiveness of the ERA is assessed by solving five well-known nonlinear problems.

**Problem 4.1.** Consider the following nonlinear FrGDE that follows:

$$\begin{array}{c} D_{\lambda }^{\varsigma }\Omega \left(\chi ,\lambda \right)+\Omega \left(\chi ,\lambda \right)D_{\chi }\Omega \left(\chi ,\lambda \right)-\Omega \left(\chi ,\lambda \right)\left(1-\Omega \left(\chi ,\lambda \right)\right)=0, \end{array}$$ (18)

where λ≥0,0≤χ≤1,0<ς≤1 with the initial condition:

$$\begin{array}{c} \Omega \left(\chi ,0\right)=e^{-\chi }. \end{array}$$ (19)

Applying the E-T to Eq (18) and using the procedure explained in Section 3, we have

$$\begin{array}{cc} B(\chi ,q) & =q^{2}e^{-\chi }-q^{\varsigma }E_{\varsigma }[E_{\varsigma }^{-1}\left[B\left(\chi ,q\right)\right]D_{\chi }E_{\varsigma }^{-1}\left[B\left(\chi ,q\right)\right]]-q^{\varsigma }E_{\varsigma }[E_{\varsigma }^{-1}\left[B\left(\chi ,q\right)\right]]+\\ & q^{\varsigma }E_{\varsigma }{\left[E_{\varsigma }^{-1}\left[B\left(\chi ,q\right)\right]\right]}^{2}. \end{array}$$ (20)

where $E_{\varsigma }\left[\Omega \left(\chi ,\lambda \right)\right]=B\left(\chi ,q\right)$ and $\Omega \left(\chi ,\lambda \right)=E_{\varsigma }^{-1}\left[B\left(\chi ,q\right)\right]$.

By using Lemma 1(iii), we get as

$$\begin{array}{c} {ℵ}_{0}\left(\chi \right)=\underset{q\to 0}{\text{lim}}(\frac{1}{q^{2}}B\left(\chi ,q\right))=\Omega \left(\chi ,0\right)=e^{-\chi }. \end{array}$$ (21)

The ERESF is defined as

$$\begin{array}{cc} ERES(\chi ,q) & =B\left(\chi ,q\right)-q^{2}e^{-\chi }-q^{\varsigma }E_{\varsigma }[E_{\varsigma }^{-1}\left[B\left(\chi ,q\right)\right]D_{\chi }E_{\varsigma }^{-1}\left[B\left(\chi ,q\right)\right]]\\ & -q^{\varsigma }E_{\varsigma }[E_{\varsigma }^{-1}\left[B\left(\chi ,q\right)\right]]+q^{\varsigma }E_{\varsigma }{\left[E_{\varsigma }^{-1}\left[B\left(\chi ,q\right)\right]\right]}^{2}. \end{array}$$ (22)

The *κ*th-truncated ERESF is expressed as follows:

$$\begin{array}{cc} E{RES}_{\kappa }\left(\chi ,q\right) & =B_{\kappa }\left(\chi ,q\right)-q^{2}e^{-\chi }-q^{\varsigma }E_{\varsigma }[E_{\varsigma }^{-1}\left[B_{\kappa }\left(\chi ,q\right)\right]D_{\chi }E_{\varsigma }^{-1}\left[B_{\kappa }\left(\chi ,q\right)\right]]\\ & -q^{\varsigma }E_{\varsigma }[E_{\varsigma }^{-1}\left[B_{\kappa }\left(\chi ,q\right)\right]]+q^{\varsigma }E_{\varsigma }{\left[E_{\varsigma }^{-1}\left[B_{\kappa }\left(\chi ,q\right)\right]\right]}^{2}. \end{array}$$ (23)

When we utilize the first truncated series $B_{1}\left(\chi ,q\right)=e^{-\chi }q^{2}+{ℵ}_{1}\left(\chi \right)q^{\varsigma +2}$ in the first $E{RES}_{1}\left(\chi ,q\right)$, we obtain the following results:

$$\begin{array}{cc} E{RES}_{1}\left(\chi ,q\right) & =q^{2}e^{-\chi }+q^{\varsigma +2}{ℵ}_{1}\left(\chi \right)-q^{2}e^{-\chi }-q^{\varsigma }\\ & E_{\varsigma }[E_{\varsigma }^{-1}\left[q^{2}e^{-\chi }+q^{\varsigma +2}{ℵ}_{1}\left(\chi \right)\right]D_{\chi }E_{\varsigma }^{-1}\left[q^{2}e^{-\chi }+q^{\varsigma +2}{ℵ}_{1}\left(\chi \right)\right]]-q^{\varsigma }\\ & E_{\varsigma }[E_{\varsigma }^{-1}\left[q^{2}e^{-\chi }+q^{\varsigma +2}{ℵ}_{1}\left(\chi \right)\right]]+q^{\varsigma }E_{\varsigma }{\left[E_{\varsigma }^{-1}\left[q^{2}e^{-\chi }+q^{\varsigma +2}{ℵ}_{1}\left(\chi \right)\right]\right]}^{2}. \end{array}$$ (24)

By solving ${\text{lim}}_{q\to 0}(\frac{1}{q^{\varsigma +2}}E{RES}_{1}\left(\chi ,q\right))=0$ for ℵ_1_(*χ*), we have ℵ_1_(*χ*) = *e*^−*χ*^. When we utilize the second truncated series $B_{2}\left(\chi ,q\right)=e^{-\chi }q^{2}+e^{-\chi }q^{\varsigma +2}+{ℵ}_{2}\left(\chi \right)q^{2\varsigma +2}$ in the second $E{RES}_{2}\left(\chi ,q\right)$, we achieve the following results:

$$\begin{array}{cc} E{RES}_{2}\left(\chi ,q\right) & =q^{2}e^{-\chi }+q^{\varsigma +2}e^{-\chi }+q^{2\varsigma +2}e^{-\chi }-q^{2}e^{-\chi }-q^{\varsigma }E_{\varsigma }[E_{\varsigma }^{-1}[q^{2}e^{-\chi }+\\ & q^{\varsigma +2}e^{-\chi }+q^{2\varsigma +2}e^{-\chi }]D_{\chi }E_{\varsigma }^{-1}\left[q^{2}e^{-\chi }+q^{\varsigma +2}e^{-\chi }+q^{2\varsigma +2}e^{-\chi }\right]]\\ & -q^{\varsigma }E_{\varsigma }[E_{\varsigma }^{-1}\left[q^{2}e^{-\chi }+q^{\varsigma +2}e^{-\chi }+q^{2\varsigma +2}e^{-\chi }\right]]+q^{\varsigma }E_{\varsigma }[E_{\varsigma }^{-1}[q^{2}e^{-\chi }+\\ & q^{\varsigma +2}e^{-\chi }+q^{2\varsigma +2}e^{-\chi }]{]}^{2}. \end{array}$$ (25)

By solving ${\text{lim}}_{q\to 0}(\frac{1}{q^{2\varsigma +2}}E{RES}_{2}\left(\chi ,q\right))=0$ for ℵ_2_(*χ*), we have ℵ_2_(*χ*) = *e*^−*χ*^. We obtained the following coefficients of series solutions for *κ* = 3, 4, 5, 6: ℵ_3_(*χ*) = *e*^−*χ*^, ℵ_4_(*χ*) = *e*^−*χ*^, ℵ_5_(*χ*) = *e*^−*χ*^, ℵ_6_(*χ*) = *e*^−*χ*^.

In this way, the expansion-form solution of Eq (18) is provided as

$$\begin{array}{c} B\left(\chi ,q\right)=e^{-\chi }+q^{\varsigma +2}e^{-\chi }+q^{2\varsigma +2}e^{-\chi }+q^{3\varsigma +2}e^{-\chi }+q^{4\varsigma +2}e^{-\chi }+q^{5\varsigma +2}e^{-\chi }+\cdots \;. \end{array}$$

By using the inverse E-T, we get an expansion solution in the original space.

$$\begin{array}{cc} \Omega (\chi ,\lambda )= & e^{-\chi }+e^{-\chi }\frac{\lambda^{\varsigma }}{\Gamma (\varsigma +1)}+e^{-\chi }\frac{\lambda^{2\varsigma }}{\Gamma (2\varsigma +1)}+e^{-\chi }\frac{\lambda^{3\varsigma }}{\Gamma (3\varsigma +1)}+e^{-\chi }\frac{\lambda^{4\varsigma }}{\Gamma (4\varsigma +1)}+\\ & e^{-\chi }\frac{\lambda^{5\varsigma }}{\Gamma (5\varsigma +1)}+\cdots . \end{array}$$ (26)

The Ex-S to Eq (18) is $\Omega \left(\chi ,\lambda \right)=e^{-\chi }E_{\varsigma }\left(\lambda^{\varsigma }\right)$. A similar result has been obtained by [36].

**Problem 4.2.** Consider the following nonlinear FrGDE:

$$\begin{array}{c} D_{\lambda }^{\varsigma }\Omega \left(\chi ,\lambda \right)+\Omega \left(\chi ,\lambda \right)D_{\chi }\Omega \left(\chi ,\lambda \right)-\Omega \left(\chi ,\lambda \right)\;\text{log}\;\eta -\Omega^{2}\left(\chi ,\lambda \right)\;\text{log}\;\eta =0, \end{array}$$ (27)

where λ≥0,0≤χ≤1,0<ς≤1 with the initial condition:

$$\begin{array}{c} \Omega \left(\chi ,0\right)=\eta^{-\chi }. \end{array}$$ (28)

Applying the E-T to Eq (27) and using the procedure explained in Section 3, we have

$$\begin{array}{cc} B(\chi ,q)= & q^{2}\eta^{-\chi }-q^{\varsigma }E_{\varsigma }[E_{\varsigma }^{-1}\left[B\left(\chi ,q\right)\right]D_{\chi }E_{\varsigma }^{-1}\left[B\left(\chi ,q\right)\right]]-\\ & q^{\varsigma }E_{\varsigma }[E_{\varsigma }^{-1}\left[B\left(\chi ,q\right)\right]]\;\text{log}\;\eta +q^{\varsigma }E_{\varsigma }{\left[E_{\varsigma }^{-1}\left[B\left(\chi ,q\right)\right]\right]}^{2}\;\text{log}\;\eta . \end{array}$$

By using Lemma 1(iii), we get as

$$\begin{array}{c} {ℵ}_{0}\left(\chi \right)=\underset{q\to 0}{\text{lim}}(\frac{1}{q^{2}}B\left(\chi ,q\right))=\Omega \left(\chi ,0\right)=\eta^{-\chi }. \end{array}$$ (29)

The ERESF is defined as

$$\begin{array}{cc} ERES(\chi ,q)= & B\left(\chi ,q\right)-q^{2}\eta^{-\chi }-q^{\varsigma }E_{\varsigma }[E_{\varsigma }^{-1}\left[B\left(\chi ,q\right)\right]D_{\chi }E_{\varsigma }^{-1}\left[B\left(\chi ,q\right)\right]]\\ & -q^{\varsigma }E_{\varsigma }[E_{\varsigma }^{-1}\left[B\left(\chi ,q\right)\right]]\;\text{log}\;\eta +q^{\varsigma }E_{\varsigma }{\left[E_{\varsigma }^{-1}\left[B\left(\chi ,q\right)\right]\right]}^{2}\;\text{log}\;\eta . \end{array}$$ (30)

The *κ*th-truncated expression for the ERESF is represented as follows:

$$\begin{array}{cc} E{RES}_{\kappa }\left(\chi ,q\right)= & B_{\kappa }\left(\chi ,q\right)-q^{2}\eta^{-\chi }-q^{\varsigma }E_{\varsigma }[E_{\varsigma }^{-1}\left[B_{\kappa }\left(\chi ,q\right)\right]D_{\chi }E_{\varsigma }^{-1}\left[B_{\kappa }\left(\chi ,q\right)\right]]\\ & -q^{\varsigma }E_{\varsigma }[E_{\varsigma }^{-1}\left[B_{\kappa }\left(\chi ,q\right)\right]]\;\text{log}\;\eta +q^{\varsigma }E_{\varsigma }{\left[E_{\varsigma }^{-1}\left[B_{\kappa }\left(\chi ,q\right)\right]\right]}^{2}\;\text{log}\;\eta . \end{array}$$ (31)

$$\begin{array}{c} B_{\kappa }\left(\chi ,q\right)=q^{2}\eta^{-\chi }+q^{\varsigma +2}\eta^{-\chi }\;\text{log}\;\eta +q^{2\varsigma +2}\eta^{-\chi }{\left(\text{log}\;\eta \right)}^{2}. \end{array}$$ (32)

When we utilize the first truncated series $B_{1}\left(\chi ,q\right)=\eta^{-\chi }q^{2}+ℵ1\left(\chi \right)q^{\varsigma +2}$ in the first $E{RES}_{1}\left(\chi ,q\right)$, we obtain the following results:

$$\begin{array}{cc} E{RES}_{1}\left(\chi ,q\right)= & q^{2}\eta^{-\chi }+q^{\varsigma +2}\eta^{-\chi }\;\text{log}\;\eta -q^{\varsigma }E_{\varsigma }[E_{\varsigma }^{-1}\left[q^{2}\eta^{-\chi }+q^{\varsigma +2}\eta^{-\chi }\;\text{log}\;\eta \right]D_{\chi }\\ & E_{\varsigma }^{-1}\left[q^{2}\eta^{-\chi }+q^{\varsigma +2}\eta^{-\chi }\;\text{log}\;\eta \right]]-q^{\varsigma }E_{\varsigma }[E_{\varsigma }^{-1}[q^{2}\eta^{-\chi }+q^{\varsigma +2}\eta^{-\chi }\\ & \text{log}\eta ]]\;\text{log}\;\eta +q^{\varsigma }E_{\varsigma }{\left[E_{\varsigma }^{-1}\left[q^{2}\eta^{-\chi }+q^{\varsigma +2}\eta^{-\chi }\;\text{log}\;\eta \right]\right]}^{2}\;\text{log}\;\eta . \end{array}$$ (33)

By solving ${\text{lim}}_{q\to 0}(\frac{1}{q^{\varsigma +2}}E{RES}_{1}\left(\chi ,q\right))=0$ for ℵ_1_(*χ*), we have ℵ_1_(*χ*) = *η*^−*χ*^ log *η*. When we utilize the second truncated series $B_{2}\left(\chi ,q\right)=\eta^{-\chi }q^{2}+q^{\varsigma +2}\eta^{-\chi }\;\text{log}\;\eta +{ℵ}_{2}\left(\chi \right)q^{2\varsigma +2}$ in the second $E{RES}_{2}\left(\chi ,q\right)$, we achieve the following results:

$$\begin{array}{cc} E{RES}_{2}\left(\chi ,q\right) & =q^{2}\eta^{-\chi }+q^{\varsigma +2}\eta^{-\chi }\;\text{log}\;\eta +q^{2\varsigma +2}{ℵ}_{2}\left(\chi \right)-q^{\varsigma }E_{\varsigma }[E_{\varsigma }^{-1}[q^{2}\eta^{-\chi }+q^{\varsigma +2}\eta^{-\chi }\\ & \text{log}\eta +q^{2\varsigma +2}{ℵ}_{2}\left(\chi \right)]D_{\chi }E_{\varsigma }^{-1}\left[q^{2}\eta^{-\chi }+q^{\varsigma +2}\eta^{-\chi }\;\text{log}\;\eta +q^{2\varsigma +2}{ℵ}_{2}\left(\chi \right)\right]]\\ & -q^{\varsigma }E_{\varsigma }[E_{\varsigma }^{-1}\left[q^{2}\eta^{-\chi }+q^{\varsigma +2}\eta^{-\chi }\;\text{log}\;\eta +q^{2\varsigma +2}{ℵ}_{2}\left(\chi \right)\right]]\;\text{log}\;\eta +q^{\varsigma }\\ & E_{\varsigma }{\left[E_{\varsigma }^{-1}\left[q^{2}\eta^{-\chi }+q^{\varsigma +2}\eta^{-\chi }\;\text{log}\;\eta +q^{2\varsigma +2}{ℵ}_{2}\left(\chi \right)\right]\right]}^{2}\;\text{log}\;\eta . \end{array}$$ (34)

By solving ${\text{lim}}_{q\to 0}(\frac{1}{q^{2\varsigma +2}}E{RES}_{2}\left(\chi ,q\right))=0$ for ℵ_2_(*χ*), we have ℵ_2_(*χ*) = *η*^−*χ*^(log *η*)^2^. We get the following coefficients of series solutions for *κ* = 3, 4, 5, 6: ℵ_3_(*χ*) = *η*^−*χ*^(log *η*)^3^, ℵ_4_(*χ*) = *η*^−*χ*^(log *η*)^4^, ℵ_5_(*χ*) = *η*^−*χ*^(log *η*)^5^, and ℵ_6_(*χ*) = *η*^−*χ*^(log *η*)^6^. In this way, the expansion-form solution of Eq (27) becomes as follows:

$$\begin{array}{cc} B(\chi ,q)= & \eta^{-\chi }+\eta^{-\chi }q^{\varsigma +2}\;\text{log}\;\eta +\eta^{-\chi }q^{2\varsigma +2}{\left(\text{log}\;\eta \right)}^{2}+\eta^{-\chi }q^{3\varsigma +2}{\left(\text{log}\;\eta \right)}^{3}+\\ & \eta^{-\chi }q^{4\varsigma +2}{\left(\text{log}\;\eta \right)}^{4}+\eta^{-\chi }q^{5\varsigma +2}{\left(\text{log}\;\eta \right)}^{5}+\cdots . \end{array}$$ (35)

By using the inverse E-T, we get an expansion solution in the original space.

$$\begin{array}{cc} \Omega (\chi ,\lambda )= & \eta^{-\chi }+\eta^{-\chi }\frac{\lambda^{\varsigma }}{\Gamma (\varsigma +1)}\;\text{log}\;\eta +\eta^{-\chi }\frac{\lambda^{2\varsigma }}{\Gamma (2\varsigma +1)}{\left(\text{log}\;\eta \right)}^{2}+\eta^{-\chi }\frac{\lambda^{3\varsigma }}{\Gamma (3\varsigma +1)}{\left(\text{log}\;\eta \right)}^{3}\\ & +\eta^{-\chi }\frac{\lambda^{4\varsigma }}{\Gamma (4\varsigma +1)}{\left(\text{log}\;\eta \right)}^{4}+\eta^{-\chi }\frac{\lambda^{5\varsigma }}{\Gamma (5\varsigma +1)}{\left(\text{log}\;\eta \right)}^{5}+\cdots . \end{array}$$ (36)

The following is the Ex-S to Eq (27):

$$\begin{array}{c} \Omega \left(\chi ,\lambda \right)=\eta^{-\chi }E_{\varsigma }\left(\lambda^{\varsigma }\;\text{log}\;\eta \right). \end{array}$$

The same solution is achieved by [36].

**Problem 4.3.** Consider the following nonlinear FrSHE:

$$\begin{array}{c} D_{\lambda }^{\varsigma }\Omega \left(\chi ,\lambda \right)+\left(1-C\right)\Omega \left(\chi ,\lambda \right)+2D_{\chi \chi }\Omega \left(\chi ,\lambda \right)+D_{\chi \chi \chi \chi }\Omega \left(\chi ,\lambda \right)-MD_{\chi \chi \chi }\Omega \left(\chi ,\lambda \right)\\ -\Omega^{2}\left(\chi ,\lambda \right)+{\left(D_{\chi }\Omega \left(\chi ,\lambda \right)\right)}^{2}=0,\;0<\varsigma \leq 1, \end{array}$$ (37)

with the initial condition:

$$\begin{array}{c} \Omega \left(\chi ,0\right)=e^{\chi }. \end{array}$$ (38)

Through the process outlined in Section 3, we have applied the E-T to Eq (37).

$$\begin{array}{cc} B(\chi ,q) & =q^{2}e^{\chi }-\left(1-C\right)q^{\varsigma }B\left(\chi ,q\right)-2q^{\varsigma }D_{\chi \chi }B\left(\chi ,q\right)-q^{\varsigma }D_{\chi \chi \chi \chi }B\left(\chi ,q\right)+Mq^{\varsigma }\\ & D_{\chi \chi \chi }B\left(\chi ,q\right)+q^{\varsigma }E_{\varsigma }{\left[E_{\varsigma }^{-1}\left[B\left(\chi ,q\right)\right]\right]}^{2}-q^{\varsigma }E_{\varsigma }{\left[D_{\chi }E_{\varsigma }^{-1}\left[B\left(\chi ,q\right)\right]\right]}^{2}. \end{array}$$ (39)

By using Lemma 1(iii), we get as

$$\begin{array}{c} {ℵ}_{0}\left(\chi \right)=\underset{q\to 0}{\text{lim}}(\frac{1}{q^{2}}B\left(\chi ,q\right))=\Omega \left(\chi ,0\right)=e^{\chi }. \end{array}$$ (40)

The ERESF is defined as

$$\begin{array}{cc} ERES(\chi ,q) & =B\left(\chi ,q\right)-q^{2}e^{\chi }+\left(1-C\right)q^{\varsigma }B\left(\chi ,q\right)+2q^{\varsigma }2pD_{\chi \chi }B\left(\chi ,q\right)+\\ & q^{\varsigma }D_{\chi \chi \chi }B\left(\chi ,q\right)-Mq^{\varsigma }D_{\chi \chi \chi }B\left(\chi ,q\right)-q^{\varsigma }E_{\varsigma }{\left[E_{\varsigma }^{-1}\left[B\left(\chi ,q\right)\right]\right]}^{2}\\ & +q^{\varsigma }E_{\varsigma }{\left[D_{\chi }E_{\varsigma }^{-1}\left[B\left(\chi ,q\right)\right]\right]}^{2}. \end{array}$$ (41)

The *κ*th-truncated ERESF is as

$$\begin{array}{cc} E{RES}_{\kappa }\left(\chi ,q\right)= & B_{\kappa }\left(\chi ,q\right)-q^{2}e^{\chi }+\left(1-C\right)q^{\varsigma }B_{\kappa }\left(\chi ,q\right)+2q^{\varsigma }2pD_{\chi \chi }B_{\kappa }\left(\chi ,q\right)+\\ & q^{\varsigma }D_{\chi \chi \chi }B_{\kappa }\left(\chi ,q\right)-Mq^{\varsigma }D_{\chi \chi \chi }B_{\kappa }\left(\chi ,q\right)-q^{\varsigma }E_{\varsigma }{\left[E_{\varsigma }^{-1}\left[B_{\kappa }\left(\chi ,q\right)\right]\right]}^{2}\\ & +q^{\varsigma }E_{\varsigma }{\left[D_{\chi }E_{\varsigma }^{-1}\left[B_{\kappa }\left(\chi ,q\right)\right]\right]}^{2}. \end{array}$$ (42)

By utilizing the *κ*th-truncated ERESF and *κ*th-series, we get the following results: ${ℵ}_{1}\left(\chi \right)=e^{\chi }\left(M+C-4\right)$, ${ℵ}_{2}\left(\chi \right)=e^{\chi }{\left(M+C-4\right)}^{2}$, ${ℵ}_{3}\left(\chi \right)=e^{\chi }{\left(M+C-4\right)}^{3}$, ${ℵ}_{4}\left(\chi \right)=e^{\chi }{\left(M+C-4\right)}^{4}$, and ${ℵ}_{5}\left(\chi \right)=e^{\chi }{\left(M+C-4\right)}^{5}$. In this way, the expansion-form solution of Eq (37) becomes as follows:

$$\begin{array}{cc} B(\chi ,q)= & e^{\chi }+e^{\chi }q^{\varsigma +2}\left(M+C-4\right)+e^{\chi }q^{2\varsigma +2}{\left(M+C-4\right)}^{2}+e^{\chi }q^{3\varsigma +2}{\left(M+C-4\right)}^{3}\\ & +e^{\chi }q^{4\varsigma +2}{\left(M+C-4\right)}^{4}+e^{\chi }q^{5\varsigma +2}{\left(M+C-4\right)}^{5}+\cdots . \end{array}$$

By using the inverse E-T, we get an expansion solution in the original space:

$$\begin{array}{cc} \Omega (\chi ,\lambda )= & e^{\chi }+e^{\chi }\frac{\lambda^{\varsigma }}{\Gamma (\varsigma +1)}\left(M+C-4\right)+e^{\chi }\frac{\lambda^{2\varsigma }}{\Gamma (2\varsigma +1)}{\left(M+C-4\right)}^{2}\\ & +e^{\chi }\frac{\lambda^{3\varsigma }}{\Gamma (3\varsigma +1)}{\left(M+C-4\right)}^{3}+e^{\chi }\frac{\lambda^{4\varsigma }}{\Gamma (4\varsigma +1)}{\left(M+C-4\right)}^{4}\\ & +e^{\chi }\frac{\lambda^{5\varsigma }}{\Gamma (5\varsigma +1)}{\left(M+C-4\right)}^{5}+\cdots . \end{array}$$

The following is the Ex-S to Eq (37) is $\Omega \left(\chi ,\lambda \right)=e^{\chi }E_{\varsigma }\left(\lambda^{\varsigma }\left(M+C-4\right)\right)$. The same solution is obtained by [44].

**Problem 4.4.** Consider the following nonlinear FrSHE that follows:

$$\begin{array}{cc} D_{\lambda }^{\varsigma }\Omega \left(\chi ,\lambda \right) & +\left(1-C\right)\Omega \left(\chi ,\lambda \right)+2D_{\chi \chi }\Omega \left(\chi ,\lambda \right)+D_{\chi \chi \chi \chi }\Omega \left(\chi ,\lambda \right)\\ & -\Omega^{2}\left(\chi ,\lambda \right)+{\left(D_{\chi }\Omega \left(\chi ,\lambda \right)\right)}^{2}=0,0<\varsigma \leq 1, \end{array}$$ (43)

with the initial condition:

$$\begin{array}{c} \Omega \left(\chi ,0\right)=e^{\chi }. \end{array}$$ (44)

Through utilizing the method discussed in Section 3 and implementing the E-T to Eq (43), we have

$$\begin{array}{cc} B(\chi ,q)= & q^{2}e^{\chi }-\left(1-C\right)q^{\varsigma }B\left(\chi ,q\right)-2q^{\varsigma }D_{\chi \chi }B\left(\chi ,q\right)-q^{\varsigma }D_{\chi \chi \chi \chi }B\left(\chi ,q\right)\\ & +q^{\varsigma }E_{\varsigma }{\left[E_{\varsigma }^{-1}\left[B\left(\chi ,q\right)\right]\right]}^{2}-q^{\varsigma }E_{\varsigma }[D_{\chi }E_{\varsigma }^{-1}{\left[B\left(\chi ,q\right)\right])}^{2}]. \end{array}$$ (45)

By using Lemma 1(iii), we get as

$$\begin{array}{c} {ℵ}_{0}\left(\chi \right)=\underset{q\to 0}{\text{lim}}(\frac{1}{q^{2}}B\left(\chi ,q\right))=F\left(\chi ,0\right)=e^{\chi }. \end{array}$$ (46)

The ERESF is defined as

$$\begin{array}{cc} ERES(\chi ,q) & =B\left(\chi ,q\right)-q^{2}e^{\chi }+\left(1-C\right)q^{\varsigma }B\left(\chi ,q\right)+2q^{\varsigma }D_{\chi \chi }B\left(\chi ,q\right)+q^{\varsigma }D_{\chi \chi \chi \chi }B\left(\chi ,q\right)\\ & -q^{\varsigma }E_{\varsigma }{\left[E_{\varsigma }^{-1}\left[B\left(\chi ,q\right)\right]\right]}^{2}+q^{\varsigma }E_{\varsigma }[D_{\chi }E_{\varsigma }^{-1}{\left[B\left(\chi ,q\right)\right])}^{2}]. \end{array}$$ (47)

The expression for the ERESF truncated to the *κ*th term is given by:

$$\begin{array}{cc} E{RES}_{\kappa }\left(\chi ,q\right) & =B_{\kappa }\left(\chi ,q\right)-q^{2}e^{\chi }+\left(1-C\right)q^{\varsigma }B_{\kappa }\left(\chi ,q\right)+2q^{\varsigma }D_{\chi \chi }B_{\kappa }\left(\chi ,q\right)+q^{\varsigma }D_{\chi \chi \chi \chi }\\ & B_{\kappa }\left(\chi ,q\right)-q^{\varsigma }E_{\varsigma }{\left[E_{\varsigma }^{-1}\left[B_{\kappa }\left(\chi ,q\right)\right]\right]}^{2}+q^{\varsigma }E_{\varsigma }[D_{\chi }E_{\varsigma }^{-1}\left[B_{\kappa }\left(\chi ,q\right)\right]{]}^{2}]. \end{array}$$ (48)

By utilizing the *κ*th-truncated ERESF and *κ*th-series, we get the following results: ${ℵ}_{1}\left(\chi \right)=e^{\chi }\left(C-4\right)$, ${ℵ}_{2}\left(\chi \right)=e^{\chi }{\left(C-4\right)}^{2}$, ${ℵ}_{3}\left(\chi \right)=e^{\chi }{\left(C-4\right)}^{3}$, ${ℵ}_{4}\left(\chi \right)=e^{\chi }{\left(C-4\right)}^{4}$, and ${ℵ}_{5}\left(\chi \right)=e^{\chi }{\left(C-4\right)}^{5}$. In this way, the expansion-form solution of Eq (43) becomes as follows:

$$\begin{array}{cc} B(\chi ,q) & =e^{\chi }+e^{\chi }q^{\varsigma +2}\left(C-4\right)+e^{\chi }q^{2\varsigma +2}{\left(C-4\right)}^{2}+e^{\chi }q^{3\varsigma +2}{\left(C-4\right)}^{3}\\ & +e^{\chi }q^{4\varsigma +2}{\left(C-4\right)}^{4}+e^{\chi }q^{5\varsigma +2}{\left(C-4\right)}^{5}+\cdots . \end{array}$$ (49)

By using the inverse E-T, we get an expansion solution in the original space:

$$\begin{array}{cc} \Omega (\chi ,\lambda ) & =e^{\chi }+e^{\chi }\frac{\lambda^{\varsigma }}{\Gamma (\varsigma +1)}\left(C-4\right)+e^{\chi }\frac{\lambda^{2\varsigma }}{\Gamma (2\varsigma +1)}{\left(C-4\right)}^{2}+e^{\chi }\frac{\lambda^{3\varsigma }}{\Gamma (3\varsigma +1)}{\left(C-4\right)}^{3}\\ & +e^{\chi }\frac{\lambda^{4\varsigma }}{\Gamma (4\varsigma +1)}{\left(C-4\right)}^{4}+e^{\chi }\frac{\lambda^{5\varsigma }}{\Gamma (5\varsigma +1)}{\left(C-4\right)}^{5}+\cdots . \end{array}$$ (50)

The Ex-S solution of Eq (43) becomes as follows:

$$\begin{array}{c} \Omega \left(\chi ,\lambda \right)=e^{\chi }E_{\varsigma }\left(\lambda^{\varsigma }\left(C-4\right)\right). \end{array}$$

A similar result has been obtained by [44].

**Problem 4.5.** Consider the following nonlinear FrFPE that follows:

$$\begin{array}{c} D_{\lambda }^{\varsigma }\Omega \left(\chi ,\lambda \right)+D_{\chi }(\frac{4}{\chi }\Omega^{2}\left(\chi ,\lambda \right)-\frac{\chi }{3}\Omega \left(\chi ,\lambda \right))-(D_{\chi \chi }\Omega^{2}\left(\chi ,\lambda \right)=0, \end{array}$$ (51)

where λ ≥ 0, 0 ≤ *χ* ≤ 1, and 0 ≤ *ς* ≤ 1, with the initial condition:

$$\begin{array}{c} \Omega \left(\chi ,0\right)=\chi^{2}. \end{array}$$ (52)

Applying the E-T to Eq (51) and using the procedure explained in Section 3, we have

$$\begin{array}{cc} B(\chi ,q) & =q^{2}\chi^{2}-D_{\chi }(\frac{4}{\chi }q^{\varsigma }E_{p}[E_{p}^{-1}\left[B_{\kappa }\left(\chi ,q\right)\right]]-\frac{\chi }{3}q^{\varsigma }B\left(\chi ,q\right))\\ & +D_{\chi \chi }(q^{\varsigma }E_{\varsigma }{\left[E_{\varsigma }^{-1}\left[B_{\kappa }\left(\chi ,q\right)\right]\right]}^{2})]. \end{array}$$ (53)

By using Lemma 1(iii), we get as

$$\begin{array}{c} {ℵ}_{0}\left(\chi \right)=\underset{q\to 0}{\text{lim}}(\frac{1}{q^{2}}B\left(\chi ,q\right))=\Omega \left(\chi ,0\right)=\chi^{2}. \end{array}$$ (54)

The ERESF is defined as

$$\begin{array}{cc} ERES(\chi ,q) & =B\left(\chi ,q\right)-D_{\chi }(\frac{4}{\chi }q^{\varsigma }E_{p}{\left[E_{p}^{-1}\left[B_{\kappa }\left(\chi ,q\right)\right]\right]}^{2}-\frac{\chi }{3}q^{\varsigma }B\left(\chi ,q\right))\\ & +D_{\chi \chi }(q^{\varsigma }E_{\varsigma }{\left[E_{\varsigma }^{-1}\left[B_{\kappa }\left(\chi ,q\right)\right]\right]}^{2})]. \end{array}$$ (55)

The expression for the ERESF truncated at the *κ*th term is as follows:

$$\begin{array}{cc} E{RES}_{\kappa }\left(\chi ,q\right) & =B_{\kappa }\left(\chi ,q\right)-q^{2}e^{\chi }+D_{\chi }(\frac{4}{\chi }q^{\varsigma }E_{p}{\left[E_{p}^{-1}\left[B_{\kappa }\left(\chi ,q\right)\right]\right]}^{2}-\frac{\chi }{3}\frac{1}{q^{\varsigma }}B\left(\chi ,q\right))\\ & +D_{\chi \chi }(q^{\varsigma }E_{\varsigma }{\left[E_{\varsigma }^{-1}\left[B_{\kappa }\left(\chi ,q\right)\right]\right]}^{2})]. \end{array}$$ (56)

By utilizing the *κ*th-truncated ERES and *κ*th series, we get the following results: ℵ_1_(*χ*) = *χ*^2^, ℵ_2_(*χ*) = *χ*^2^, ℵ_3_(*χ*) = *χ*^2^, ℵ_4_(*χ*) = *χ*^2^, *and* ℵ_5_(*χ*) = *χ*^2^. In this way, the expansion-form solution of Eq (51) is provided as

$$\begin{array}{c} B\left(\chi ,q\right)=\chi^{2}q^{\varsigma +2}+\chi^{2}q^{2\varsigma +2}+\chi^{2}q^{3\varsigma +2}+\chi^{2}q^{4\varsigma +2}+\chi^{2}q^{5\varsigma +2}+\ldots . \end{array}$$

By using the inverse E-T, we get an expansion solution in the original space:

$$\begin{array}{cc} \Omega (\chi ,\lambda ) & =\chi^{2}+\chi^{2}\frac{\lambda^{\varsigma }}{\Gamma (\varsigma +1)}+\chi^{2}\frac{\lambda^{2\varsigma }}{\Gamma (2\varsigma +1)}+\chi^{2}\frac{\lambda^{3\varsigma }}{\Gamma (3\varsigma +1)}\\ & +\chi^{2}\frac{\lambda^{4\varsigma }}{\Gamma (4\varsigma +1)}+\chi^{2}\frac{\lambda^{5\varsigma }}{\Gamma (5\varsigma +1)}+\cdots . \end{array}$$ (57)

The Ex-S of Eq (51) becomes as $\Omega \left(\chi ,\lambda \right)=\chi^{2}E_{\varsigma }\left(\lambda \right)$. A similar result has been obtained by [52].

## 5 Graphical and numerical results

In this section, we analyze the numerical and graphical results of the Ex-Ss and App-Ss for the five nonlinear problems presented in the fourth section of this research study. To assess the accuracy of the ERA, we employ various error functions, such as the Abs-E, Rel-E, and Res-E functions. Delineating the errors in the approximate solutions is essential since ERA provides an approximation expressed in terms of an infinite FPS.

NPFDEs serve as powerful tools for modeling complex systems that exhibit memory and non-local effects. However, Ex-Ss to NPFDEs are often difficult to obtain, necessitating the use of approximate methods. Understanding the Abs-E, Rel-E, and Res-E functions is crucial for assessing the accuracy and reliability of these approximate methods for solving NPFDEs. By quantifying these errors, researchers can make informed decisions regarding the choice of numerical techniques and refine existing approaches to improve the accuracy of solutions to NPFDEs. Additionally, investigating error propagation mechanisms aids in developing more robust algorithms for various applications in science and engineering.

First, we present some useful notation for Ex-S and App-S, along with formulas for error functions, which we utilize in this section to analyze the reliability and correctness of our approach.

$$\begin{array}{c} \Omega (\chi ,\lambda )\approx \Omega^{\kappa }\left(\chi ,\lambda \right),\;\kappa =1,\;2,\;3,\cdots , \end{array}$$

where Ω(*χ*, λ) and Ω^*κ*^(*χ*, λ) denote the Ex-S and App-S of the problems obtained by ERA.

Abs-E plays a crucial role in quantifying the accuracy of these App-Ss and is indispensable for validating and refining numerical techniques. In the context of NPFDEs, the Abs-E represents the magnitude of the difference between the App-S obtained through approximate methods and the Ex-S, if available. It is calculated as the absolute difference between the Ω^*κ*^(*χ*, λ) and the Ω(*χ*, λ) at each point in the solution domain. Smaller Abs-E values indicate higher accuracy in the approximation. The Abs-E is defined as follows:

$$\begin{array}{c} Abs.E^{\kappa }\left(\chi ,\lambda \right)=|\Omega \left(\chi ,\lambda \right)-\Omega^{\kappa }\left(\chi ,\lambda \right)|,\;\kappa =1,\;2,\;3,\cdots , \end{array}$$

where the Ω(*χ*, λ) is represented by *Abs*.*E*^*κ*(*χ*, λ)^, which is the Abs-E for the *κ*th-step App-S. When *κ* increases to infinity, it frequently happens that Abs.E^*κ*^(*χ*, λ) gets decreasing, eventually decreasing almost to zero.

The Rel-E serves as a powerful tool for evaluating the effectiveness of the approach that generates App-Ss. It is calculated as the ratio of the Abs-E to the magnitude of the Ex-S at each point within the solution domain. This provides valuable insights into the degree of alignment between the App-S and the behavior of the Ex-S. A smaller Rel-E indicates higher accuracy in the approximation. Mathematically, it is defined as follows:

$$\begin{array}{c} Rel.E^{\kappa }\left(\chi ,\lambda \right)=\frac{|\Omega \left(\chi ,\lambda \right)-\Omega^{\kappa }\left(\chi ,\lambda \right)|}{\left|\Omega (\chi ,\lambda )\right|},\;\kappa =1,\;2,\;3,\cdots , \end{array}$$

where the Rel-E for the *κ*th-step App-S is represented by Rel.E^*κ*^(*χ*, λ) for the Ex-S Ω(*χ*, λ). In fact, it often happens that as *κ* goes to infinity, Rel.E^*κ*^(*χ*, λ) gets ever smaller until it almost reaches zero.

The Res-E for differential equations is a critical measure used to evaluate the accuracy of approximate solutions. When solving differential equations, especially those that are nonlinear or involve fractional derivatives, exact solutions are often unattainable. Thus, approximate methods are employed, and the Res-E helps quantify how well these App-Ss satisfy the original differential equations. The residual error is the difference between the Ex-S and the App-S when substituted back into the differential equation. It essentially measures the discrepancy or error introduced by the approximation. The importance of Res-E is as follows:

i. Accuracy Assessment: The Res-E provides a quantitative measure of the accuracy of the App-S. A smaller Res-E indicates a more accurate approximation.
ii. Method Evaluation: By comparing the Res-E of different methods, one can determine which method is more effective for solving a particular differential equation.
iii. Improvement and Optimization: Analyzing the Res-E can help identify areas where the approximation can be improved, leading to better numerical or analytical techniques.

**Fig 1:**
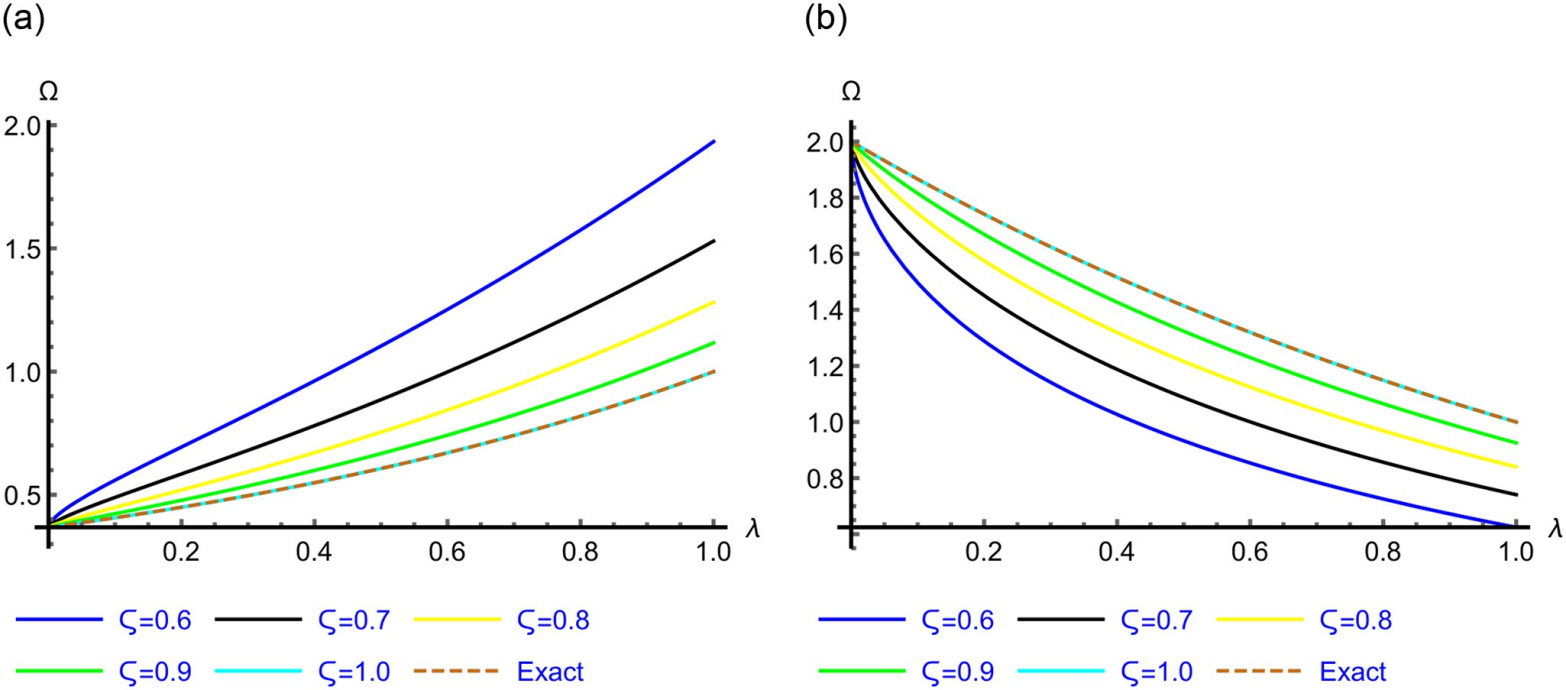
The 2D diagrams of App-Ss and Ex-Ss for various levels of *ς* in the range λ ∈ [0, 1.0] at *χ* = 1.0 for (a) Problem 4.1, and *χ* = 1.0 with *η* = 0.5 for (b) Problem 4.2, respectively.

**Fig 2:**
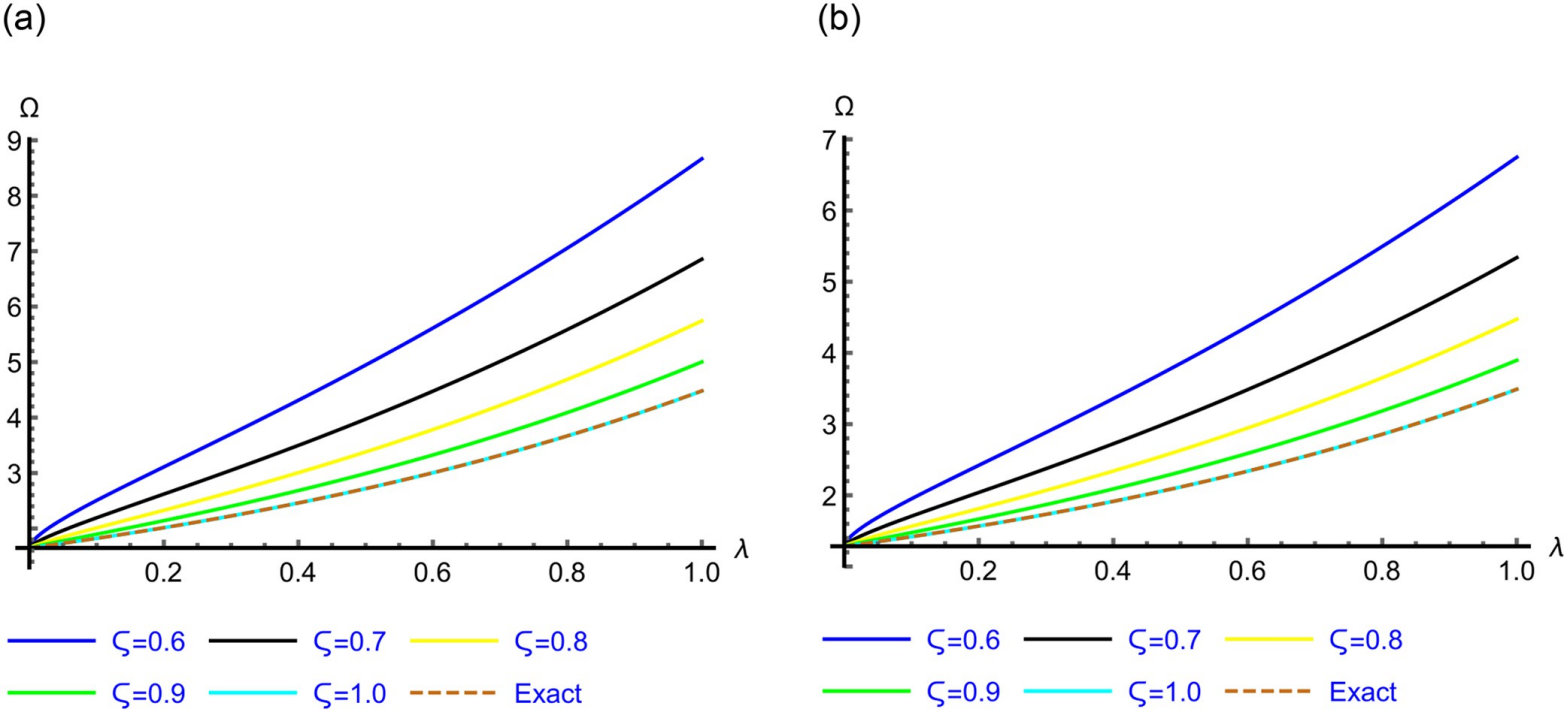
The 2D diagrams of App-Ss and Ex-Ss for various levels of *ς* in the range λ ∈ [0, 1.0] at *χ* = 0.5 when C=3.0 and M=2.0 for (a) Problem 4.3, and at *χ* = 0.25 when C=5.0 for (b) Problems 4.4.

**Fig 3:**
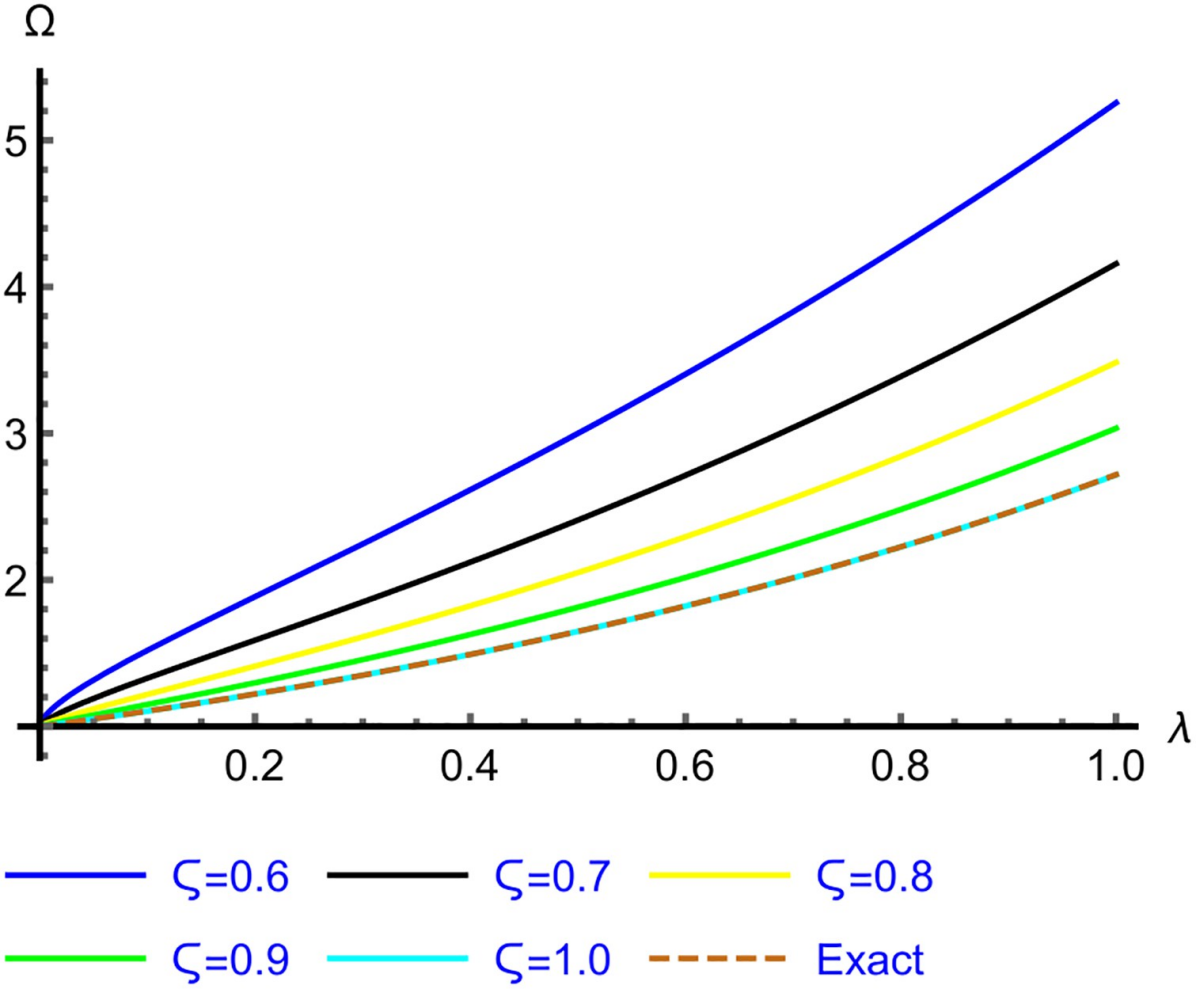
The 2D diagrams of App-S and Ex-S for various levels of *ς* in the range λ ∈ [0, 1.0] at *χ* = 1.0 for Problem 4.5.

The 2D graphs of the App-Ss obtained from five iterations and the Ex-Ss derived by ERA for *ς* = 0.6, 0.7, 0.8, 0.9 and 1.0 are depicted in Figs 1–3 for Problems 4.1–4.5. These graphs illustrate how, as *ς* → 1.0, the App-Ss converge to the Ex-Ss. The interaction between the App-Ss and Ex-Ss when *ς* = 1.0 demonstrates the accuracy of the proposed approach.

**Fig 4:**
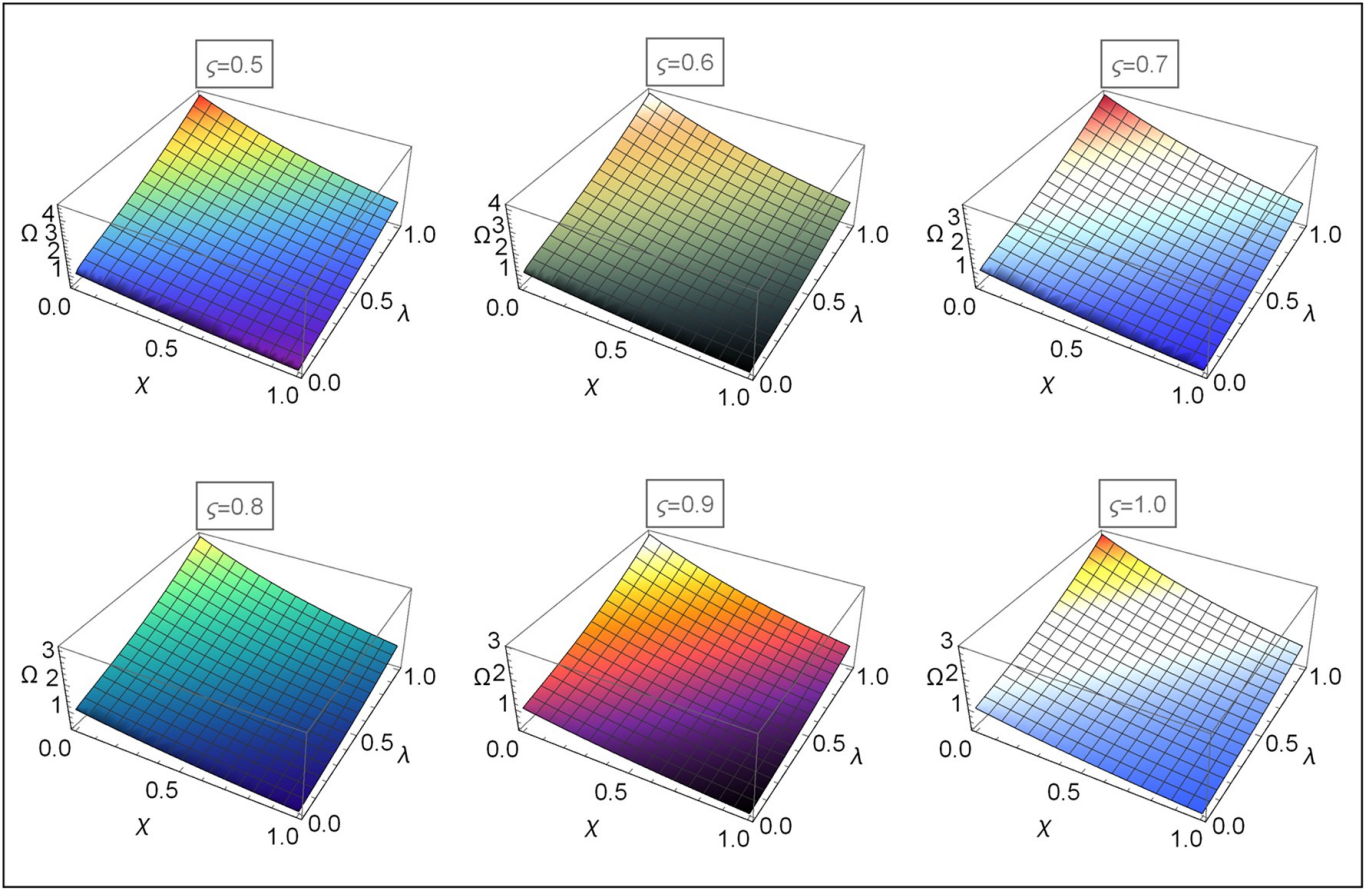
The 3D diagrams of App-Ss and Ex-S for various levels of *ς* in the range λ ∈ [0, 1.0] and *χ* ∈ [0, 1.0] with *η* = 0.5 for Problem 4.1.

**Fig 5:**
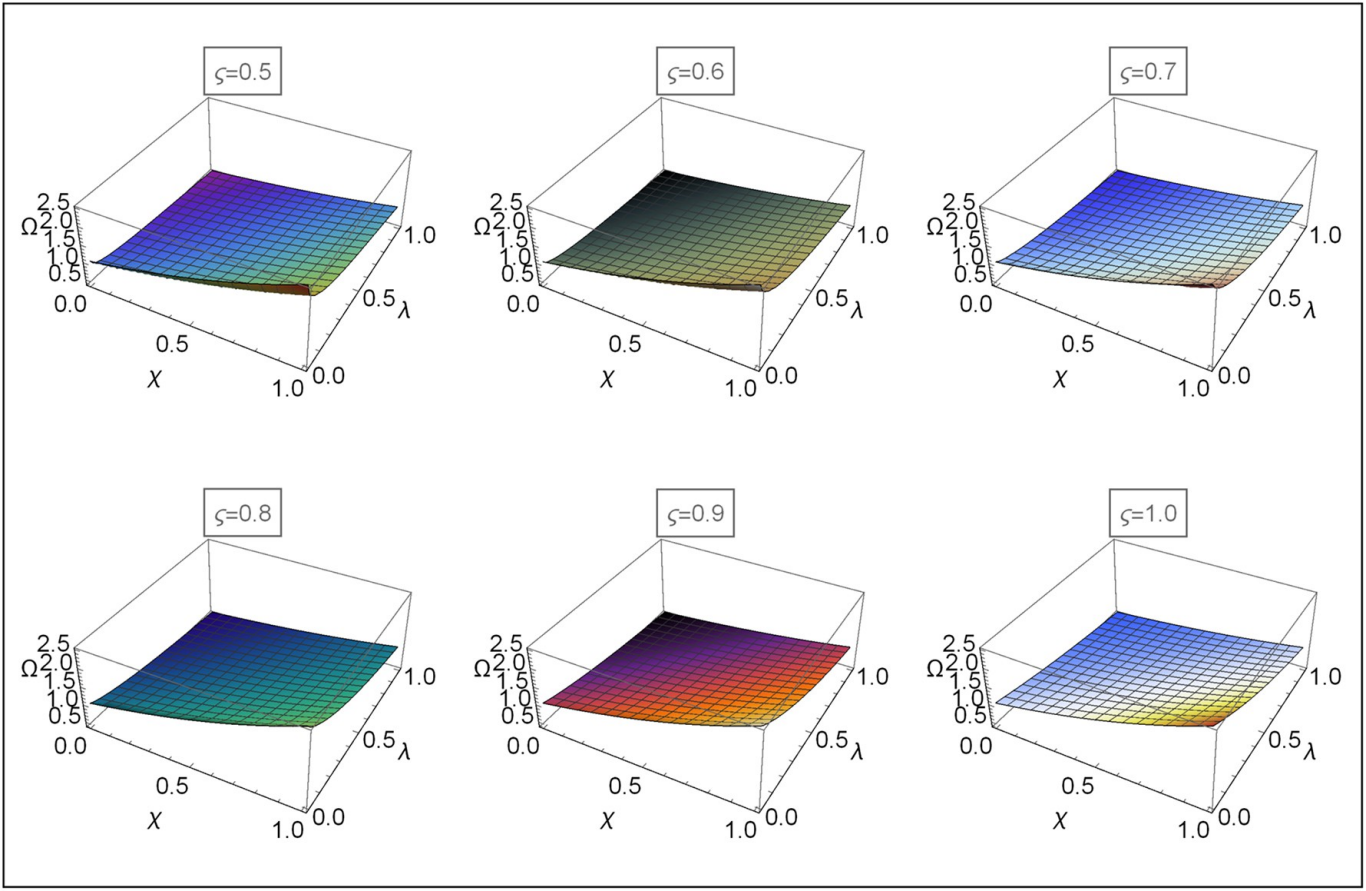
The 3D diagrams of App-Ss and Ex-S for various levels of *ς* in the range λ ∈ [0, 1.0] and *χ* ∈ [0, 1.0] with *η* = 0.5 for Problem 4.2.

**Fig 6:**
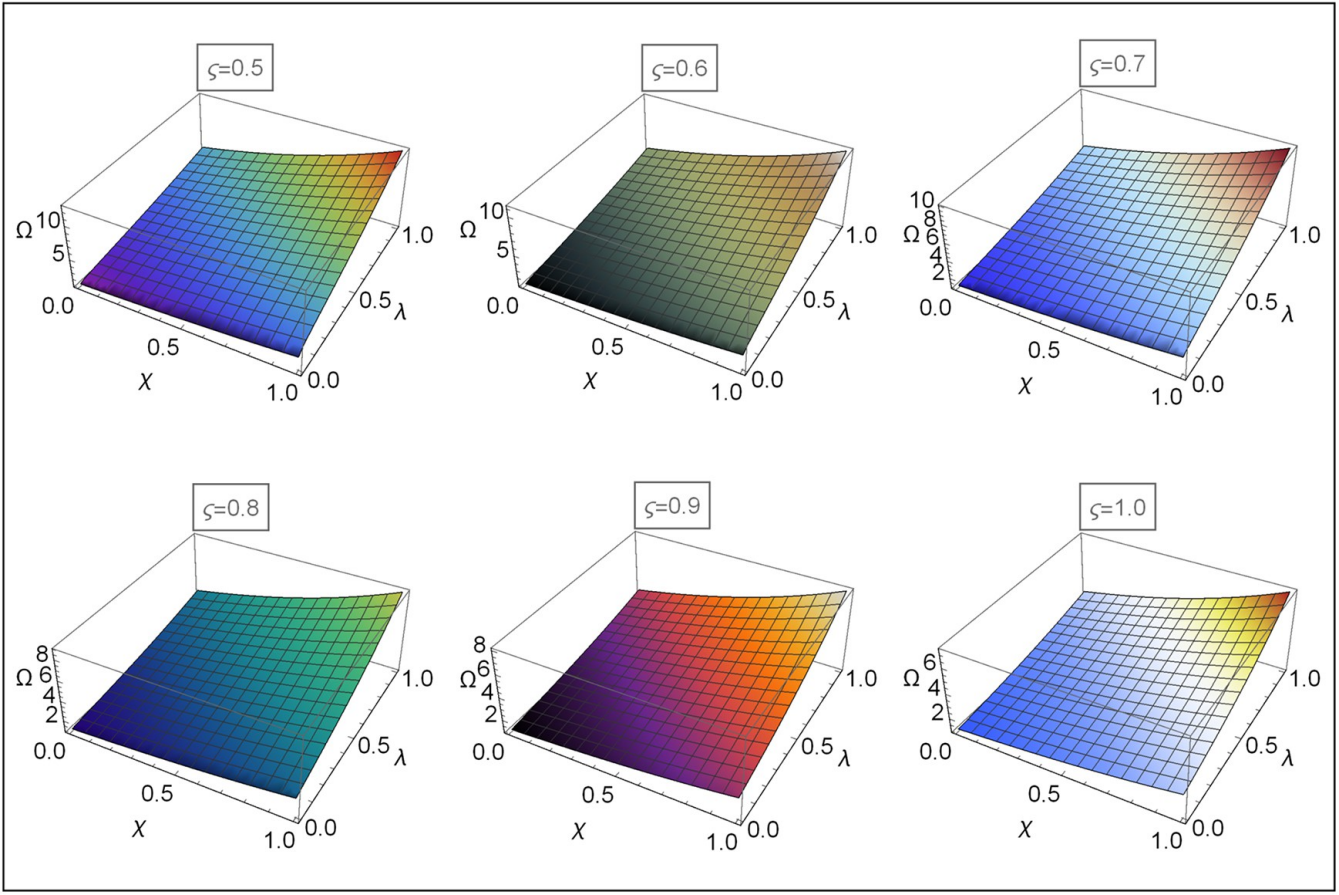
The 3D diagrams of App-Ss and Ex-S for various levels of *ς* in the range λ ∈ [0, 1.0] and *χ* ∈ [0, 1.0] when C=3.0 and M=2.0 for Problem 4.3.

**Fig 7:**
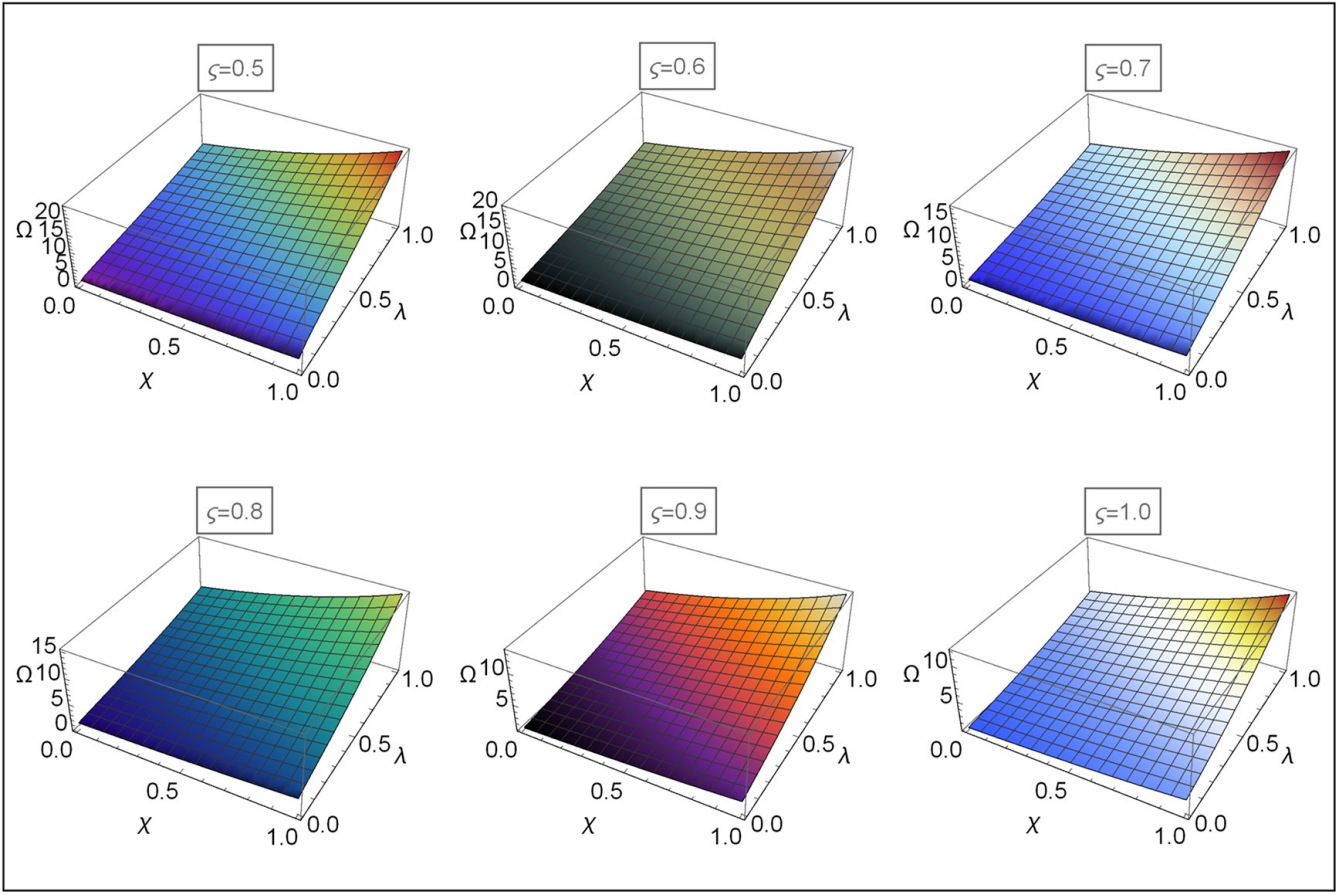
The 3D diagrams of App-Ss and Ex-S for various levels of *ς* in the range λ ∈ [0, 1.0] and *χ* ∈ [0, 1.0] when C=5.0 for Problem 4.4.

**Fig 8:**
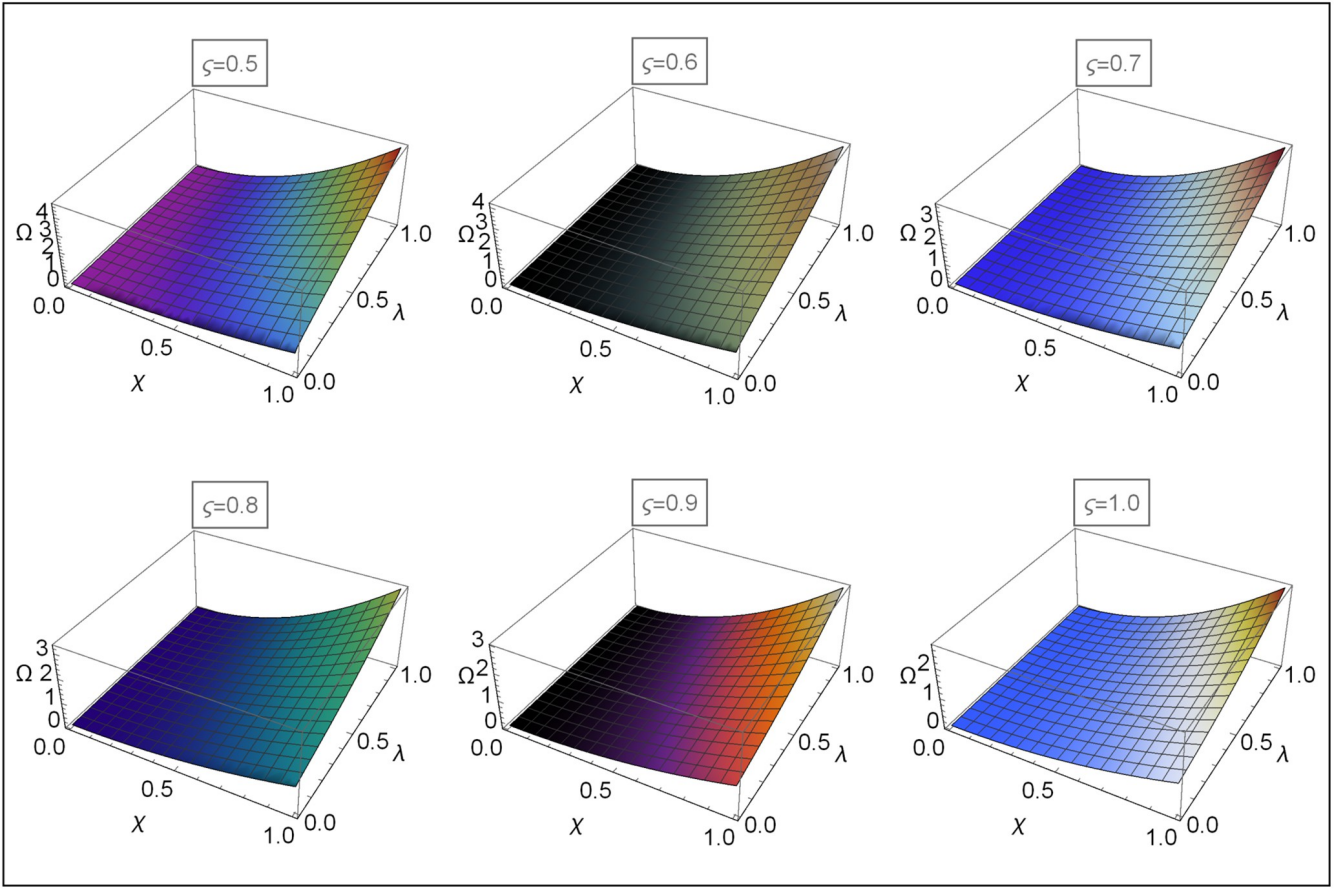
The 3D diagrams of App-Ss and Ex-S for various levels of *ς* in the range λ ∈ [0, 1.0] and *χ* ∈ [0, 1.0] for Problem 4.5.

Figs 4–8 for Problems 4.1–4.5 display the 3D graphs of the App-Ss obtained from five iterations and the Ex-Ss determined by ERA for *ς* = 0.5, 0.6, 0.7, 0.8, 0.9, and 1.0. These graphs demonstrate how the App-Ss converge to the Ex-Ss as *ς* approaches 1.0. The interaction between the App-Ss and Ex-Ss when *ς* = 1.0 illustrates the accuracy of the proposed method.

**Fig 9:**
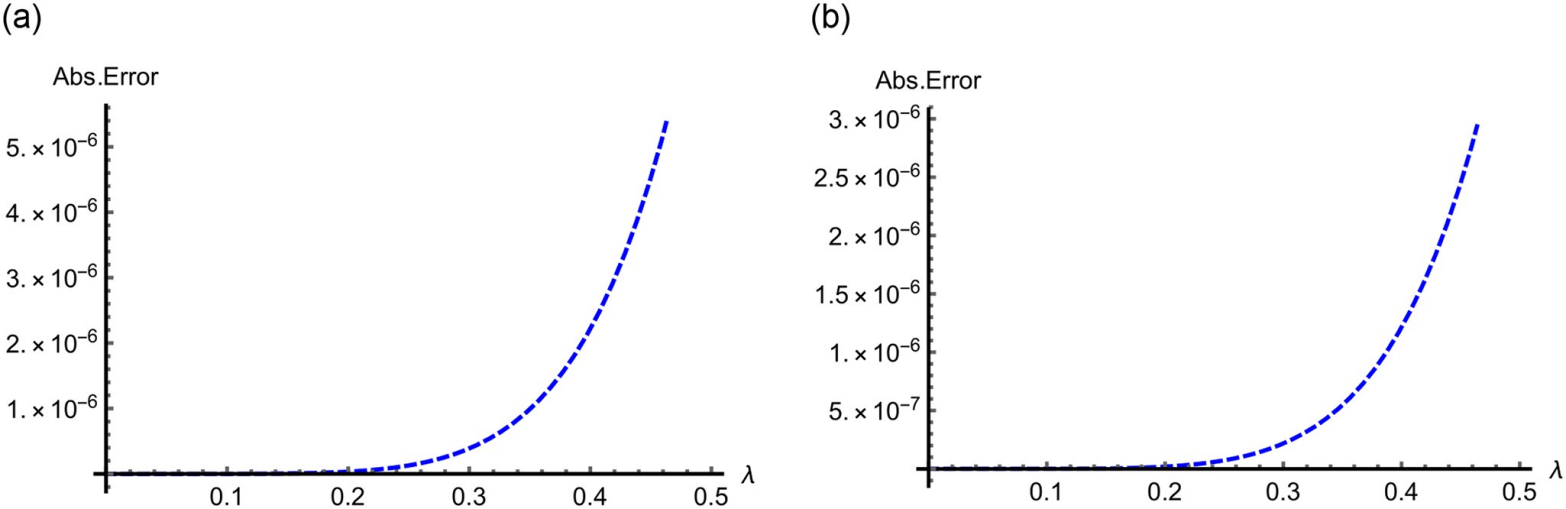
Two-dimensional Abs-E graphs in the range of λ ∈ [0, 0.5] between the fifth step App-S and Ex-S for *ς* = 1.0 when *χ* = 1.0 for (a) Problems 4.1, and when *χ* = 1.0 with *u* = 0.5 for (b) Problem 4.2.

**Fig 10:**
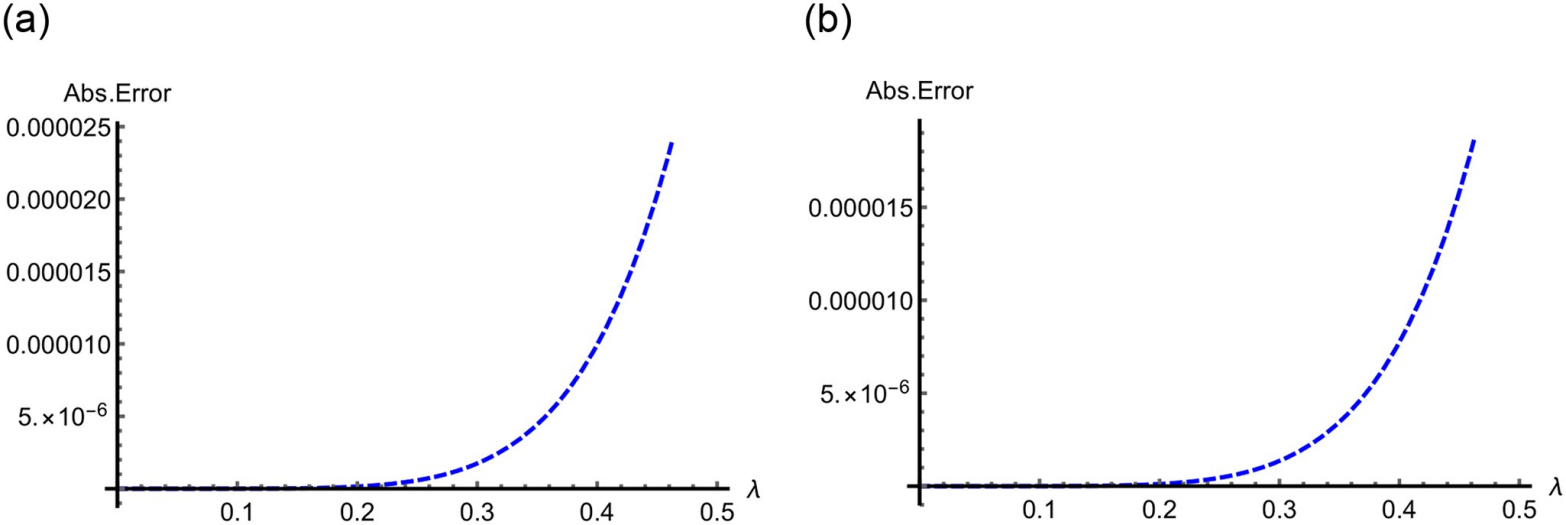
Two-dimensional Abs-E graphs in the range of λ ∈ [0, 0.5] between the fifth step App-S and Ex-S for ς=1.0 when *χ* = 0.5 with C=3.0 and M=2.0 for (a) Problems 4.3, and when *χ* = 0.25 with C=5.0 for (b) Problem 4.4.

**Fig 11:**
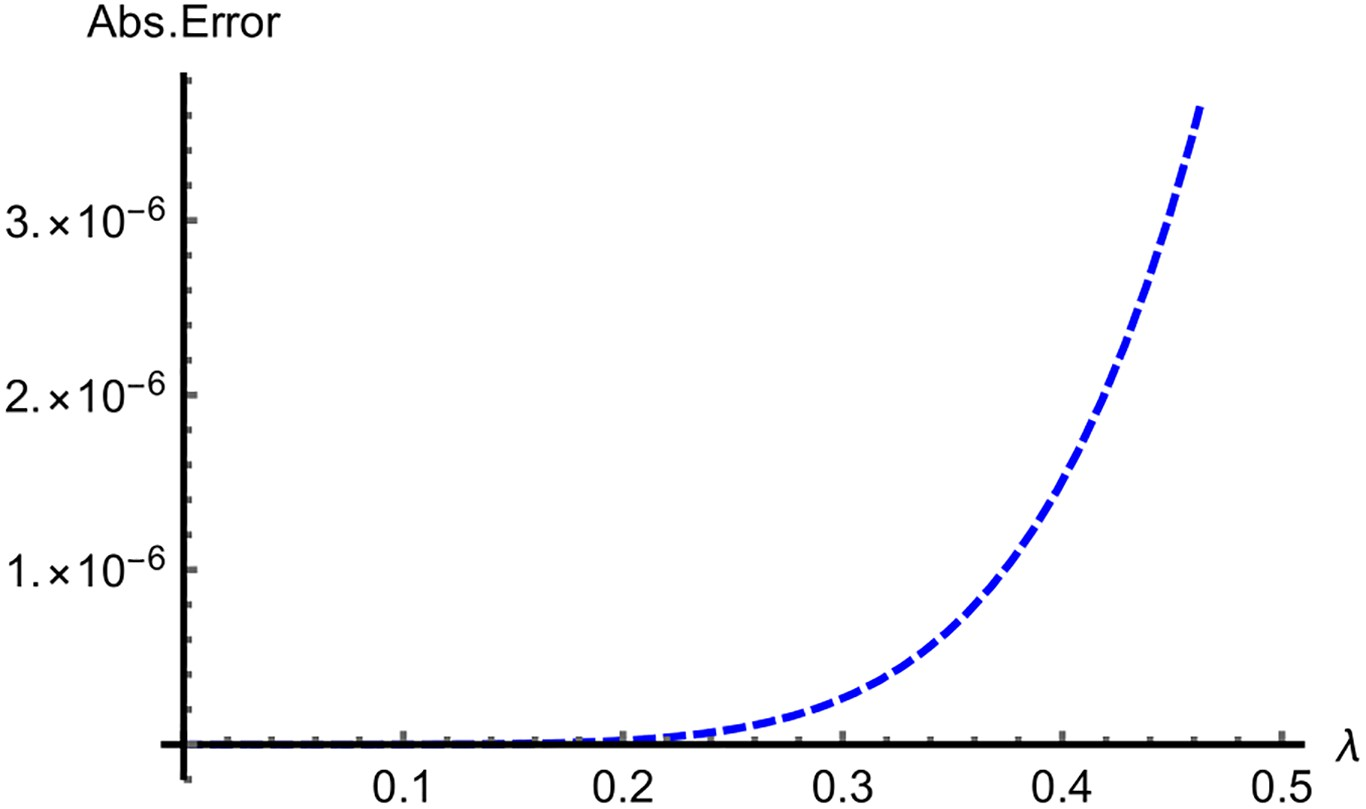
The 2D diagrams of Abs-E in the range of λ ∈ [0, 0.5] between the fifth step App-S and Ex-S for ς=1.0 when ϰ=0.5 for Problems 4.5.

In Figs 9–11 for Problems 4.1–4.5, 2D curves are employed to compare the App-Ss and Ex-Ss in terms of Abs-E. The comparative analysis reveals a high degree of similarity between the fifth-step App-Ss and the Ex-Ss. The Abs-E is presented on the graphs to demonstrate the excellent precision of ERA.

The Abs-E and Rel-E for specified locations between the Ex-Ss and fifth-order App-Ss derived by ERA in Problems 4.1–4.5 at *ς* = 1.0 are presented in Tables 1–5. These tables demonstrate that the App-Ss and Ex-Ss are nearly in agreement, confirming the accuracy of ERA. Tables 6–10 display the Res-E for the fifth App-Ss over the interval λ ∈ [0, 0.5], as obtained by ERA for Problems 4.1–4.5 at *ς* = 0.6, 0.7, 0.8, 0.9, 1.0. From these tables, it is observed that the Res-E for all problems in the fifth-step App-Ss is very small. The findings presented in this section, depicted in both graphs and tables, demonstrate that ERA is a useful and effective technique for solving NPFDEs, requiring fewer calculations and iterations.

**Table 1:** The Abs-E and Rel-E at varying values of λ when *χ* = 1.0 for Problem 4.1.

λ	Ω(1, λ)	Ω^5^(1, λ)	*Rel.Error*	*Abs.Error*
0.1	0.4065696598023920	0.40656965922226396	1.274898753901053 ×10^−9^	5.1833515257726500 ×10^−10^
0.2	0.44932896417792534	0.44932893045864280	7.490854461965522×10^−8^	3.3658578757478350 ×10^−8^
0.3	0.49658530385011157	0.49658491473094050	7.834715728350320×10^−7^	3.8906046900821780 ×10^−7^
0.4	0.54881163614963420	0.54880941742245950	0.000004042683174163541	0.0000022186715670224901
0.5	0.60653065976384690	0.60652206824385460	0.000014164937322248202	0.0000085914687788513080
0.6	0.67032004608091900	0.67029400002754420	0.000038856078151207712	0.0000260460080950819501
0.7	0.74081822071924920	0.74075152752212270	0.000090026348884621653	0.0000666931595951769701
0.8	0.81873075310563430	0.81857982618677230	0.000184342521204015304	0.0001509268912096573002
0.9	0.90483741805123990	0.90452661098567340	0.000343494913109170505	0.0003108070502861881603
1.0	1.00000000000000000	0.99940581535119140	0.000594184648808560006	0.0005941846488085600004

**Table 2:** The Abs-E and Rel-E at varying values of λ when *χ* = 1.0 at *η* = 0.5 for Problem 4.2.

λ	Ω(1, λ)	Ω^5^(1, λ)	*Rel.Error*	*Abs.Error*
0.1	1.8660659827685684	1.8660659830736148	1.634703356286456×10^−10^	1.050464325582425 ×10^−10^
0.2	1.741101107259536	1.7411011265922482	1.110372741975478×10^−8^	1.933271231990829 ×10^−8^
0.3	1.624504574631055	1.624504792712471	1.342448585040598×10^−7^	1.180814160368527 ×10^−7^
0.4	1.5157153529530853	1.5157165665103982	8.006492372491392×10^−7^	0.000001213557312862434
0.5	1.414208977153438	1.4142135623730951	0.000003242239912745257	0.000004585219657071704
0.6	1.3194943489638191	1.3195079107728942	0.000010277929343474892	0.000013561809075079978
0.7	1.1884431012121797	1.2311444133449163	0.034684243107370935000	0.042701312132736646000
0.8	1.148623576037244	1.148698354997035	0.000065098865568772420	0.000074778959791022000
0.9	1.0716232682975848	1.0717734625362931	0.000140136179853620200	0.000150194238708323270
1.0	0.9997199820546389	1.000000000000000	0.000280017945361144000	0.000280017945361144000

**Table 3:** The Abs-E and Rel-E at varying values of λ and *χ* with C=3.0 and M=2.0 for Problem 4.3.

λ	Ω(1, λ)	Ω^5^(1, λ)	*Rel.Error*	*Abs.Error*
(0.03,0.03)	1.06183654654432	1.06183654654535	9.865985960312 ×10^−13^	1.0476064460362 ×10^−12^
(0.13,0.13)	1.29693007888701	1.29693008666577	5.9977915677191 ×10^−9^	7.7787163377252 ×10^−9^
(0.23,0.23)	1.58407371746601	1.58407398499448	1.6888630985918 ×10^−7^	2.6752840986965 ×10^−7^
(0.33,0.33)	1.93478971675811	1.93479233440203	0.000001352932710503	0.0000026176438372431
(0.43,0.43)	2.36314632122551	2.36316069370579	0.000006081888669505	0.0000143724802472712
(0.53,0.53)	2.88631445113781	2.88637098926795	0.000019587963696605	0.0000565381301527101
(0.63,0.63)	3.52524252279690	3.52542148736538	0.000050764020450205	0.0001789645684802111
(0.73,0.73)	4.30547337653070	4.30595952834520	0.000112902086352814	0.0004861518145009562
(0.83,0.83)	5.25813191201622	5.25931084444689	0.000224161009976689	0.0011789324306725742
(0.93,0.93)	6.42111750626145	6.42373677142913	0.000407747898283598	0.0026192651676772981

**Table 4:** The Abs-E and Rel-E at varying values of λ and *χ* with C=3.0 for Problem 4.4.

λ	Ω(1, λ)	Ω^5^(1, λ)	*Rel.Error*	*Abs.Error*
(0.05,0.05)	1.1051709153149	1.1051709180756	2.497951209498 ×10^−9^	2.7606630315091 ×10^−9^
(0.15,0.15)	1.3498580535708	1.3498588075760	5.585807849316 ×10^−7^	7.5400519228274 ×10^−7^
(0.25,0.25)	1.6487103698322	1.6487212707001	0.00000661171056119	0.000010900867837948
(0.35,0.35)	2.0136867849099	2.0137527074704	0.00003273617475320	0.000065922560541498
(0.45,0.45)	2.4593426274569	2.4596031111569	0.00010590476928346	0.000260483700015967
(0.55,0.55)	3.0033668369534	3.0041660239464	0.00026602624043272	0.000799186992986200
(0.65,0.65)	3.6672236017846	3.6692966676192	0.00056497634897884	0.002073065834591769
(0.75,0.75)	4.4769175156003	4.4816890703380	0.00106467777278576	0.004771554737725836
(0.85,0.85)	4.4769175156003	4.4816890703380	0.00106467777278576	0.004771554737725836
(0.95,0.95)	6.6661756508830	6.6858944422792	0.00294931240186632	0.019718791396183377

**Table 5:** The Abs-E and Rel-E at varying values of λ when *χ* = 1.0 for Problem 4.5.

λ	Ω(1, λ)	Ω^5^(1, λ)	*Rel.Error*	*Abs.Error*
0.1	0.27629272951891193	0.27629272916666664	1.274898869421280 ×10^−9^	3.522452884929805 ×10^−10^
0.2	0.30535068954004246	0.30535066666666666	7.490854479152386 ×10^−8^	2.287337580453297 ×10^−8^
0.3	0.33746470189400080	0.33746443750000000	7.834715729382329 ×10^−7^	2.643940008040246 ×10^−7^
0.4	0.37295617441031760	0.37295466666666666	0.000004042683174006208	0.000001507743650930315
0.5	0.41218031767503205	0.41217447916666666	0.000014164937322380167	0.000005838508365385575
0.6	0.45552970009762720	0.45551200000000003	0.000038856078151216220	0.000017700097627193490
0.7	0.50343817686761920	0.50339285416666660	0.000090026348884752570	0.000045322700952588060
0.8	0.55638523212311700	0.55628266666666670	0.000184342521204030960	0.000102565456450265380
0.9	0.61490077778923750	0.61468956250000000	0.000343494913109226050	0.000211215289237509650
1.0	0.67916666672122200	0.67916666666666670	0.000393494913109226050	0.000291666666666667000

**Table 6:** The Res-E for the Problem 4.1 at various values of *ς* with specific values of *λ* in the interval of [0, 0.5] with *χ* = 1.0.

λ	*ς* = 0.6	*ς* = 0.7	*ς* = 0.8	*ς* = 0.9	*ς* = 1.0
0.1	0.0862136	0.0400950	0.0174227	0.0058774	3.0656620 ×10^−8^
0.2	0.1555370	0.0758171	0.0346282	0.0123075	9.8101184 ×10^−7^
0.3	0.2284715	0.1146656	0.0539696	0.0197834	7.4495586 ×10^−6^
0.4	0.3071385	0.1577112	0.0759680	0.0285120	3.1392371 ×10^−5^
0.5	0.3924850	0.2055702	0.1010150	0.0386948	9.5801931 ×10^−5^

**Table 7:** The Res-E for problem 4.2 at various values of *ς* with specific values of λin the interval of [0, 0.5], when *χ* = 1.0 and *u* = 0.5.

λ	*ς* = 0.6	*ς* = 0.7	*ς* = 0.8	*ς* = 0.9	*ς* = 1.0
0.1	0.1840312	0.1001817	0.0485237	0.0176771	3.2303145 ×10^−6^
0.2	0.2471696	0.1463037	0.0769433	0.0303745	5.1258358 ×10^−5^
0.3	0.2854440	0.1770869	0.0975785	0.0402774	2.5733540 ×10^−4^
0.4	0.3105766	0.1986929	0.1128573	0.0478413	8.0648017 ×10^−4^
0.5	0.3273033	0.2138209	0.1238968	0.0532125	1.9522787 ×10^−4^

**Table 8:** The Res-E Problem 4.3 at various of *ς* with specific values of λin the interval of [0, 0.5] with *χ* = 1.0, C=3.0, and M=2.0.

λ	*ς* = 0.6	*ς* = 0.7	*ς* = 0.8	*ς* = 0.9	*ς* = 1.0
0.1	0.3103751	0.2962643	0.1287374	0.0434284	2.2652348 ×10^−7^
0.2	0.5727935	0.5602172	0.2558702	0.0909410	7.2487515 ×10^−6^
0.3	0.6210501	0.3116934	0.3987844	0.1461813	5.5045207 ×10^−5^
0.4	0.6348892	0.4287034	0.5613320	0.2106770	2.3196004 ×10^−4^
0.5	0.6934889	0.5587979	0.7464059	0.2859184	7.0788589 ×10^−4^

**Table 9:** The Res-E for Problem 4.4 at various values of *ς* with specific values of λin the interval of [0, 0.5], when *χ* = 1.0 and C=5.0.

λ	*ς* = 0.6	*ς* = 0.7	*ς* = 0.8	*ς* = 0.9	*ς* = 1.0
0.1	0.3103751	0.2962643	0.1287374	0.0434284	2.2652348 ×10^−7^
0.2	0.5727935	0.5602172	0.2558702	0.0909410	7.2487515 ×10^−6^
0.3	0.6210501	0.3116934	0.3987844	0.1461813	5.5045207 ×10^−5^
0.4	0.6348892	0.4287034	0.5613320	0.2106770	2.3196004 ×10^−4^
0.5	0.6934889	0.5587979	0.7464059	0.2859184	7.0788589 ×10^−4^

**Table 10:** The Res-E for Problem 4.5 at various values of *ς* with specific values of λin the interval of [0, 0.5], when *χ* = 1.0.

λ	*ς* = 0.6	*ς* = 0.7	*ς* = 0.8	*ς* = 0.9	*ς* = 1.0
0.1	0.2343530	0.1089895	0.0473598	0.0159764	8.3333331 ×10^−8^
0.2	0.4227935	0.2060929	0.0941293	0.0334553	2.6666666 ×10^−8^
0.3	0.6210501	0.3116934	0.1467045	0.0537771	0.000020249991
0.4	0.8348892	0.4287034	0.2065025	0.0775037	0.000085333331
0.5	0.9934889	0.5587979	0.2745873	0.1051835	0.000260416661

## 6 Conclusions

In this study, we established solutions to nonlinear problems by employing a straightforward approach known as ERA. The correctness of ERA is assessed through an examination of absolute, relative, and residual errors presented in both numerical and graphical representations. It is observed that the App-Ss rapidly approach the Ex-Ss, as evidenced by the evaluation of 2D and numerical data across various fractional-order values. The numerical and graphical findings confirm the notable precision and effectiveness of ERA.

Four key features distinguish the ERA from other series of solution methods that researchers commonly utilize to solve problems. One advantage of this approach is its independence from any form of physical parametric assumptions concerning the problem, enabling its application to solve both weakly and severely nonlinear problems. This mitigates certain limitations previously associated with perturbation techniques. Secondly, the ERA operates without the necessity of Adomian and He’s polynomials to solve nonlinear problems, requiring only a minimal number of calculations for solving NPFDEs. As a result, it surpasses Adomian decomposition and homotopy techniques by a significant margin. The ERA presents a swift and straightforward approach to determining coefficients for the FPS. Unlike certain techniques like homotopy perturbation, variational iteration, and Adomian decomposition, which necessitate integration, while the residual FPS method requires derivatives, both of which pose challenges in fractional scenarios when determining series coefficients, the ERA merely relies on the concept of the limit at zero. Furthermore, unlike traditional analytic approximation methods, the ERA can yield expansion solutions for NPFDEs devoid of perturbation, linearization, or discretization. Following this analysis, we have established that our novel approach is both efficient and accurate.

## 7 Future directions

In the future, we intend to employ ERA to solve various nonlinear fractional models emerging in biological systems. Additionally, we will modify our method in the sense of conformable fractional derivatives and apply it to solve these models accordingly.
