# Correction: A novel technique using integral transforms and residual functions for nonlinear partial fractional differential equations involving Caputo derivatives

**DOI:** 10.1371/journal.pone.0321323

**Published:** 2025-03-27

**Authors:** Zareen A. Khan, Muhammad Bilal Riaz, Muhammad Imran Liaqat, Ali Akgül

The Funding statement for this paper is incorrect. The correct Funding statement is as follows: This article has been produced with the financial support of the European Union under the REFRESH Research Excellence for Region Sustainability and High-tech Industries project number CZ.10.03.01/00/22 003/0000048 via the Operational Programme Just Transition. This research is funded by Princess Nourah bint Abdulrahman University Researchers Supporting Project number (PNURSP2024R8), Princess Nourah bint Abdulrahman University, Riyadh, Saudi Arabia.
